# Correlating Radiomic Features of Heterogeneity on CT with Circulating Tumor DNA in Metastatic Melanoma

**DOI:** 10.3390/cancers12123493

**Published:** 2020-11-24

**Authors:** Andrew B. Gill, Leonardo Rundo, Jonathan C. M. Wan, Doreen Lau, Jeries P. Zawaideh, Ramona Woitek, Fulvio Zaccagna, Lucian Beer, Davina Gale, Evis Sala, Dominique-Laurent Couturier, Pippa G. Corrie, Nitzan Rosenfeld, Ferdia A. Gallagher

**Affiliations:** 1 Department of Radiology, University of Cambridge, Cambridge CB2 0QQ, UK; lr495@cam.ac.uk (L.R.); la399@cam.ac.uk (D.L.); Jeriespaolo.Zawaideh@addenbrookes.nhs.uk (J.P.Z.); rw585@medschl.cam.ac.uk (R.W.); f.zaccagna@mail.utoronto.ca (F.Z.); lb795@medschl.cam.ac.uk (L.B.); es220@cam.ac.uk (E.S.); fag1000@cam.ac.uk (F.A.G.); 2 Cancer Research UK Cambridge Centre, University of Cambridge, Cambridge CB2 0RE, UK; davina.gale@cruk.cam.ac.uk (D.G.); nitzan.rosenfeld@cruk.cam.ac.uk (N.R.); 3 Imaging Department, Cambridge University Hospitals NHS Foundation Trust, Cambridge CB2 0QQ, UK; 4 Cancer Research UK Cambridge Institute, University of Cambridge, Cambridge CB2 0RE, UK; jcmw3@cam.ac.uk (J.C.M.W.); Dominique-Laurent.Couturier@cruk.cam.ac.uk (D.-L.C.); 5 Department of Oncology, Cambridge University Hospitals NHS Foundation Trust, Cambridge CB2 0QQ, UK; pippa.corrie@addenbrookes.nhs.uk

**Keywords:** liquid biopsy, circulating tumor DNA, tumor heterogeneity, tumor imaging, radiomics

## Abstract

**Simple Summary:**

The analysis of circulating tumor DNA (ctDNA) concentrations in blood plasma and the radiomic analysis of tumor images (i.e., quantification of textural features on medical imaging) have both been used to provide information about cancer progression. The purpose of this study was to assess a link between these two different modalities in order to determine whether results from one can be used to predict outcomes from the other. The results show that radiomics features can predict ctDNA levels in patients with metastatic melanoma even when controlling for confounding influences such as tumor volume. This establishes the potential for new biomarkers of tumor progression that could combine the specificity of ctDNA assays with the high-resolution spatial information obtained by imaging.

**Abstract:**

Clinical imaging methods, such as computed tomography (CT), are used for routine tumor response monitoring. Imaging can also reveal intratumoral, intermetastatic, and interpatient heterogeneity, which can be quantified using radiomics. Circulating tumor DNA (ctDNA) in the plasma is a sensitive and specific biomarker for response monitoring. Here we evaluated the interrelationship between circulating tumor DNA mutant allele fraction (ctDNA*maf*), obtained by targeted amplicon sequencing and shallow whole genome sequencing, and radiomic measurements of CT heterogeneity in patients with stage IV melanoma. ctDNA*maf* and radiomic observations were obtained from 15 patients with a total of 70 CT examinations acquired as part of a prospective trial. 26 of 39 radiomic features showed a significant relationship with log(ctDNA*maf*). Principal component analysis was used to define a radiomics signature that predicted ctDNA*maf* independent of lesion volume. This radiomics signature and serum lactate dehydrogenase were independent predictors of ctDNA*maf*. Together, these results suggest that radiomic features and ctDNA*maf* may serve as complementary clinical tools for treatment monitoring.

## 1. Introduction

Detection of circulating tumor DNA (ctDNA) in the plasma is a promising tool for treatment monitoring of cancer patients and is an area of intensive research interest. Fragments of DNA are released either as a consequence of tumor cell death, or are shed from rapidly proliferating cancer cells [1]. The presence of tumor-specific mutations allows ctDNA to be distinguished from the circulating cell-free DNA derived from normal cells, and the sensitivity for detecting these can be enhanced by targeting patient-specific mutations sequenced from individual tumor biopsies [2,3]. The fractional concentration of a given somatic mutation in a background of wild-type fragments at the same nucleotide position is frequently used as a metric to quantify ctDNA, and is termed the variant or mutant allele fraction (ctDNA*maf*). ctDNA is a powerful, minimally invasive tool for real-time sampling of multifocal clonal evolution and can be used to assess changes in disease burden over time [4]. Recent developments in sequencing technologies have made the detection and quantification of ctDNA feasible and practicable for translation into routine clinical practice [5,6]. Plasma ctDNA*maf* has been shown to strongly correlate with total tumor burden in a number of tumor types and may provide earlier prediction of clinical response compared to traditional biomarkers [3,7,8,9,10]. Changes in ctDNA have been shown to be a sensitive and specific way of monitoring patient response to both chemotherapy and radiotherapy [11,12]. Despite the advantages of this approach, morphological imaging techniques, such as computed tomography (CT), remain the standard of care to monitor treatment response and form the basis of the Response Evaluation Criteria In Solid Tumors (RECIST), which are used in many clinical trials [13].

An important unanswered clinical question is how the use of ctDNA may provide additional information to complement conventional imaging approaches to improve patient management. In contrast to circulating biomarkers, imaging probes spatial tumor heterogeneity within and between lesions, and between patients [14,15]. Imaging also provides information on temporal changes in this spatial heterogeneity during natural disease progression and in response to a particular intervention, such as drug treatment. Radiomics is an emerging field within radiology that quantitatively assesses this image heterogeneity and can reveal patterns that are not apparent on visual inspection [16]. These approaches have been used to mine medical imaging data in a wide range of studies to assess for clinically useful predictor variables [17]. For example, radiomic metrics have been used in cancer studies to relate tumor image features with clinical biomarkers and survival outcomes [18,19]. Recent studies have also shown that radiomic techniques can be used to provide imaging descriptors of molecular phenotypes in tumors. There is increasing evidence that some of these quantitative metrics of heterogeneity may have a biological basis [8,18].

The primary aim of this study was to identify the extent to which quantitative radiomic features on CT can predict the ctDNA mutant allele fraction measured in plasma samples taken contemporaneously in patients with metastatic melanoma. The rationale underlying this hypothesis is that tumor heterogeneity may reflect underlying biological characteristics such as cell death or rapid proliferation that may predispose towards the release of ctDNA [20]. A secondary goal was to determine how much additional unique information each technique provides, and therefore the potential benefit in undertaking both methods simultaneously as part of a multimodal integrative cancer care approach. Although plasma ctDNA levels have been shown to correlate with overall tumor burden [10,21,22], imaging is usually a more accurate method for quantifying this and may be more sensitive than ctDNA in the context of low tumor volume. This study compared the interrelationship between ctDNA burden and a range of commonly used radiomic metrics of heterogeneity measured on CT, in patients receiving systemic therapy. Metastatic melanoma was chosen as an exemplar given the high tumor mutational burden generally present in this cancer type [23]. Fifteen patients receiving routine systemic therapy and regular CT response assessments, underwent serial ctDNA measurements (comprising targeted amplicon sequencing, TAm-Seq, and/or shallow whole genome sequencing, sWGS) as part of the MelResist prospective observational study (https://crukcambridgecentre.org.uk/trials/melresist). The aim of the study was to see if radiomic features were related to or could independently predict ctDNA*maf* concentrations observations of melanoma lesions over time and throughout treatment.

## 2. Results

**Figure 1:**
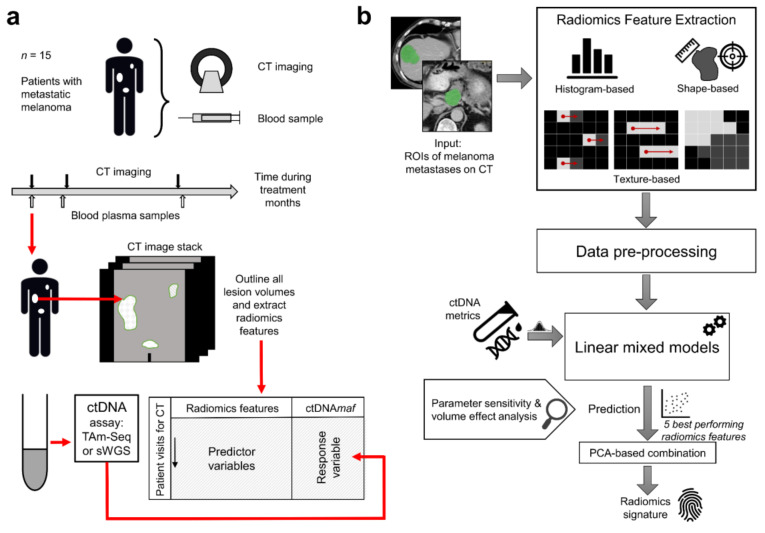
Workflow showing the study design. (**a**) Fifteen patients with metastatic melanoma were studied. Computed tomography (CT) imaging was performed throughout routine systemic therapy with concurrent blood sampling for ctDNA analysis. ctDNA was analyzed using TAm-Seq targeting *BRAF* V600 (chr7:140453136) or *NRAS* Q61 (chr1:115256530) mutations and/or shallow whole genome sequencing analyzing copy number changes using ichorCNA. Tumor regions of interest were outlined on CT and analyzed for radiomic features (shape, histogram, and texture). (**b**) The radiomic features were used to predict ctDNA*maf*: data were preprocessed using a *z*-score and/or other transformations, before linear mixed models were used in the prediction process. Patient dependence, parameter sensitivities (e.g., the effect of outliers), and the independent effect of lesion volume were all controlled for in the analysis. Principal component analysis (PCA) was used to select the best performing features to derive a radiomics signature for ctDNA*maf*.

**Figure 2:**
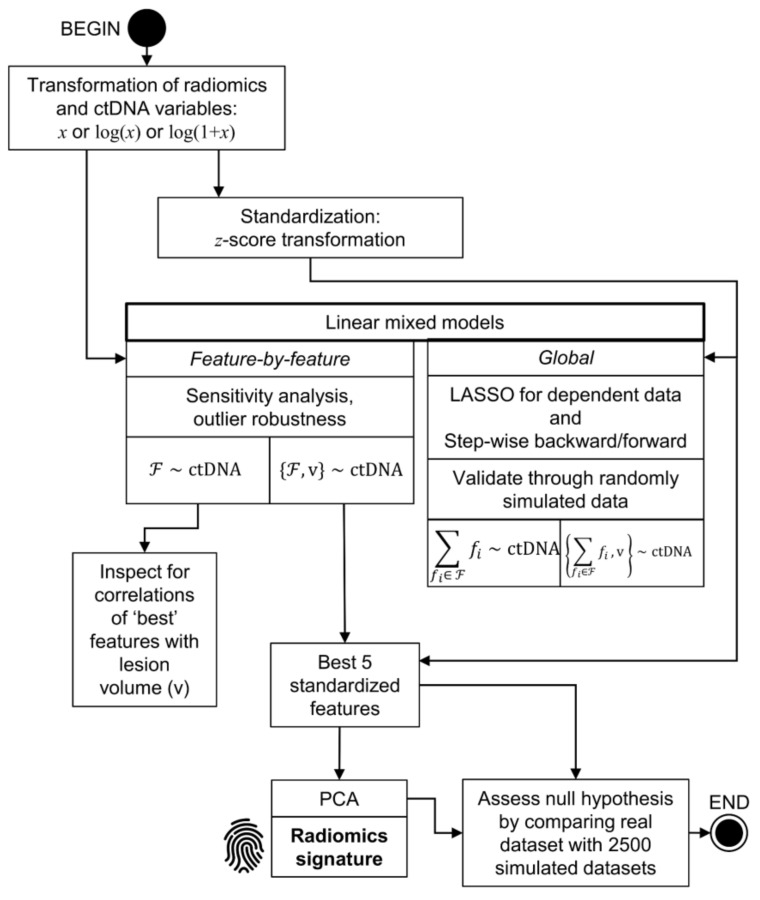
Flow-chart illustrating the detailed statistical analysis methods. The radiomic feature variables and the ctDNA*maf* response variable were both transformed for optimal statistical comparison. In most cases, including that of ctDNA*maf*, the logarithmic transform proved the most effective approach. For some of the further analyses (e.g., LASSO, other global models, and PCA), the features were standardized using a *z*-score transformation. Mixed linear models were then applied either feature-by-feature or globally using a LASSO or stepwise model for dependent data, with the features (F) used to predict ctDNA*maf* as the response variable. For the feature-by-feature models, a sensitivity analysis was carried out to examine the robustness of the modeling approaches to outliers. The models were validated through the use of simulated random data. Each modelling process was repeated with lesion volume (*v*) explicitly written into the model in order to examine the effect of the radiomic models in addition to the predictive value of lesion volume alone. Finally, the best five features yielded by the feature-by-feature linear mixed models, controlling for volume effects, were selected and subjected to a PCA. This resulted in a radiomics signature for prediction of log(ctDNA*maf*). The efficacy of this signature was assessed against that of 2500 simulated datasets, which allowed for the evaluation of the null hypothesis, i.e., that there was no significant predictive effect of the radiomic features upon ctDNA*maf*.

A flow-chart summarizing the methodology for this study is shown in Figure 1 and the strategy for statistical analysis is displayed in Figure 2.

Radiomic and ctDNA*maf* observations were derived from the lesion data and blood samples collected at time-points before, during, and after differing treatment regimes, in 15 metastatic melanoma patients (details shown in Table 1). One patient (P12) did not have usable ctDNA measurements and was not included in the analysis. In instances of blood samples that were acquired on different dates to that of the CT imaging, an interpolated mutant allele fraction was calculated from the samples closest in date to the imaging using linear interpolation. The median of the absolute interval between blood-sampling and imaging was 10 (inter-quartile range: 4, 34) days.

ctDNA*maf* measurements were acquired by either shallow whole genome sequencing (sWGS) using ichorCNA or tagged amplicon deep sequencing (TAm-Seq) targeting genomic regions in *BRAF* and *NRAS* genes (BRAF V600 (chr7:140453136) (C > T) or NRAS Q61 (chr1:115256530) (G > T). These differing measures were compared by using plasma samples collected from individual patients on the same day. There was good agreement between the two measures of ctDNA*maf* with a concordance correlation coefficient (CCC [24]) and 95% confidence interval of 0.64 (0.37, 0.81), confirming that the use of both approaches was appropriate for this study (Figure S1).

Table S1 shows the original, noninterpolated ctDNA data acquired through both sWGS and TAm-Seq; Table S2 shows the ctDNA*maf* values interpolated at the CT imaging time-points.

### 2.1. Change in the Volume of Lesions over Time and Radiomic Feature Extraction

**Figure 3:**
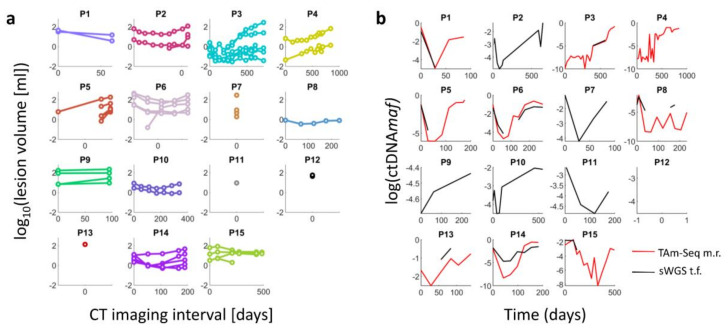
(**a**) Comparison of lesion volume for individual lesions in each subject measured on CT against date. Day 0 represents the date of first imaging. These graphs show considerable variation in tumor growth between patients and across time points. The largest lesion in each case showed volume variations which reflected standard RECIST 1.1 measurements. (**b**) ctDNAmaf by days elapsed from baseline measurement. Red line shows TAm-Seq mutation rate; black line shows shallow whole genome tumor fraction.

Figure 3 shows tumor volume plotted against time for all lesions in all patients, and demonstrates significant intertumoral and interpatient heterogeneity.

Radiomic features were calculated for the largest lesion in each patient. The largest lesion was defined as that with the greatest volume at any time-point and was tracked across imaging visits. This lesion in each case showed volume changes over time that corresponded with the overall RECIST 1.1 outcomes (see Table 1). Table 1 also shows the baseline fraction of total tumor volume, summed over imaged lesions only, which was accounted for by the largest lesion.

Radiomic features were selected from the set of shape, histogram, and texture measurements reported by the analysis software employed (LIFEx v3.40, Laboratory of Translational Imaging in Oncology, Orsay, France) [25]. Figure S2 displays the relationship between each radiomic feature and log(ctDNA*maf*) after the scale transformation used to linearize their relationship with the response variable (ctDNA*maf*), decrease heteroscedasticity, and minimize the effect of outliers. Inspection of the feature scatterplots showed that (i) observations of the same feature on the same patients tended to cluster together, suggesting some level of within-participant dependence; (ii) the assumption of bivariate normality for log(ctDNA*maf*) when paired with each feature seemed reasonable; (iii) there were outliers, justifying the use of a robust estimator (which down-weighted observations considered as outliers) as a sensitivity analysis.

### 2.2. Comparison of Radiomic Features and ctDNAmaf without Controlling for Lesion Volume

Previous studies have shown a correlation between radiomic features and tumor volume [9,10,21,22]. We therefore analyzed our data using two distinct approaches—with and without adjusting for lesion volume—to establish that any observed relationship between ctDNA*maf* and radiomic features was not simply a reflection of the underlying relationship with lesion volume (as measured by the continuous variable in cubic millimetres, and not to the categorized description of volume summarized in Table 1.)

The analysis of the relationship between the 39 extracted radiomic features and log(ctDNA*maf*) without correcting for lesion volume was first undertaken by performing a feature-by-feature analysis by means of random intercept mixed linear models (MLM) with log(ctDNA*maf*) as the response (i.e., the variable being predicted). In these models the feature of interest was included as a fixed effect predictor and the patient was a random effect, to take into account the within-patient dependence. Figure S3 shows the marginal R-squared (*y*-axis) and the *p*-values with a false discovery rate (FDR) multiplicity correction for a combined set of real and random features (*x*-axis), color-coded by feature type, for all feature-by-feature mixed linear models. The majority of the real features (26 of 39) showed a significant relationship with log(ctDNA*maf*) after a multiplicity correction. The ability of the multiplicity correction to distinguish genuine from nongenuine relationships between features and ctDNA*maf* was assessed by considering an additional set of 100 randomly generated features. Only one of these random features was selected by the model, indicating that the multiplicity correction performed well. The most predictive features were able to explain around 45% of the measured variance of the log(ctDNA*maf*) values.

Table 2 reports the Wald t-statistics, corresponding *p*-values with and without a FDR multiplicity correction, and marginal R-squared estimates for the most significant of a combined set of real and random features (the complete data are shown in Table S3). Interestingly, lesion volume correlated very strongly with ctDNA*maf* and was one of the features showing the highest significance levels (*p* = 4.2 × 10^−8^). Figure S4 shows the relationship between the radiomic features that showed a significant correlation with log(ctDNA*maf*). It is notable that many of the radiomic features strongly correlate with each other. Robust analyses led to similar conclusions, indicating that outliers did not have a significant impact on the estimation (Figure S5).

Subsequently, global analysis was performed by means of two model selection procedures suitable for data with intrapatient dependence (least absolute shrinkage and selection operator, LASSO, for dependent data; stepwise models) to define the set of radiomic features best able to predict log(ctDNA*maf*). These analyses considered log(ctDNA*maf*) as the response variable and the set of 39 radiomic features on the transformed scale as predictors. Both model selection procedures selected the same set of features as the best predictors: *LRHGE* (long-run high gray level emphasis), *GLNUz* (gray level nonuniformity zone length) and *StdDev* (standard deviation).

### 2.3. Comparison of Radiomic Features and ctDNAmaf Controlling for Lesion Volume

Given the known relationship between ctDNA*maf* and lesion volume [10,21,22], a secondary analysis was performed to determine the influence of volume on the correlations described above between radiomic features and ctDNA*maf*. The ability of the 38 radiomic features (all features except volume) to predict log(ctDNA*maf*) when lesion volume was included in the model was investigated. The relationship between each feature and lesion volume is shown in Figure S6. The results showed this relationship is often strong and linear (for example *GLNUr*) but sometimes quadratic (see feature *LZE* for example), suggesting that many features are highly dependent on lesion volume and therefore have a similar ability to predict ctDNA*maf*.

Table 3 lists the Wald t-statistics, corresponding *p*-values with and without FDR multiplicity correction, for a combined set of real and random features, and marginal R-squared estimates for the 20 best models relating radiomic features to ctDNA*maf* when including lesion volume in the model (see Table S4 for the full results). None of these showed a statistically significant predictor after a Benjamini–Hochberg multiplicity correction was performed, suggesting that in this dataset each feature alone does not have additional predictive power to determine ctDNA*maf* above lesion volume. Figure S7 shows the marginal R-squared and adjusted *p*-value corresponding to each feature-by-feature fitted model. The marginal R-squared improved when controlling for lesion volume in feature-by-feature analyses although estimates of randomly generated features often matched those of real features, indicating that the results corresponding to the latter could have been obtained by chance alone.

Given that no single feature could predict ctDNA*maf* when including lesion volume in the model with a multiplicity correction applied, principal component analysis (PCA) was performed on the five standardized features with the highest predictive value, defined as those with the smallest *p*-values. The coefficients of the first dimension for this PCA were used to define a radiomics signature as shown in Equation (1).

(1) $$\begin{array}{lllll} \left|\log(\mathrm{ctDNA}maf)\right| & ≅ & [ & +0.323\times z((\mathrm{Correlation})) & \\ & & & ‒0.528\times z(\log(\mathrm{GLNUz})) & \\ & & & +0.438\times z((\mathrm{StdDev})) & \\ & & & +0.383\times z(\log(\log(\mathrm{LGRE})+9)) & \\ & & & +0.527\times z(\log(\mathrm{Coarseness})) & ] \end{array}$$

In Equation (1), *z*(∙) and log(∙) denote a *z*-score standardization and the natural logarithmic transform, respectively. *Correlation* represents the linear dependency of gray-levels in the gray-level correlation matrix (*GLCM*); *StdDev* is the standard deviation of gray-levels in the lesion gray-level histogram; *GLNUz* is the gray-level nonuniformity for zones and measures the nonuniformity of the gray-levels of the homogeneous zones within the lesion images; *Coarseness* is a neighborhood gray level difference matrix feature representing the level of spatial rate of change in image intensity; *LGRE* is the low gray-level run-emphasis derived from the gray-level run-length matrix (*GLRLM*). (see Table S5 for a key to feature names.)

Figure S8 shows an example of feature maps generated within a large lesion for the five variables included in the radiomics signature. The texture features are themselves heterogeneous within the lesion and across features indicating the wide range of spatial information included within the signature itself.

**Figure 4:**
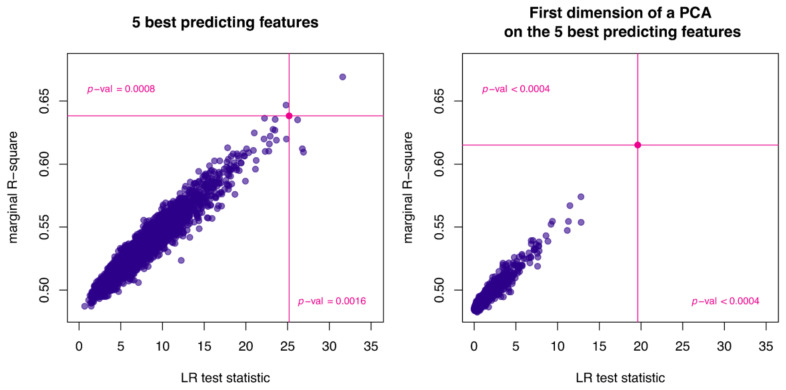
The relationship between the marginal R-squared and likelihood ratio statistics obtained when using the five most predictive features directly (**left**) or using the radiomics signature described in Equation (1) (**right**). The statistics derived from the real features are shown as pink lines, those from simulated features are shown as 2500 blue dots corresponding to 2500 simulated sets of random predictors (as described in Section 4). The proportion of blue dots in the upper right quadrant of each plot is less than 1% of the total and therefore the null hypothesis, that the observed dataset of radiomic features has no predictive power for the response variable log(ctDNA*maf*), can be rejected.

Figure 4 shows the relationship between the marginal R-squared (*y*-axis) and likelihood ratio test (LRT) statistics (*x*-axis) obtained when using the five best features each as separate predictor terms in the model (left) or through the signature described above (right), in comparison with a model incorporating lesion volume alone. In each plot, the pink dot corresponds to the R-squared and LRT statistics obtained when using the best set of five real features; the blue dots correspond to datasets composed of random features generated with similar codependencies as the real data but no correlation with log(ctDNA*maf*). In both analyses, the probability of observing a greater marginal R-squared (proportion of points above the horizontal pink line) or a greater LRT statistics (proportion of points right of the vertical pink line) than those generated with random features is smaller than 1% and consequently the null hypothesis of the real data-set not being predictive of log(ctDNA*maf*) can be rejected at the 1% significance level. Therefore, these results demonstrate that the five radiomic features in Equation (1) significantly predict ctDNA*maf* independently of lesion volume, whether directly or through the signature defined above.

### 2.4. Analysis of the Associations between ctDNAmaf, the Derived Radiomics Signature, and Serum LDH Levels

In addition, we assessed the interrelationship between ctDNA*maf*, radiomic features, and conventional plasma biomarkers of cell death that may be a surrogate measure of the cellular release of ctDNA fragments as a consequence of tumor cell death [1]. Plasma levels of the enzyme lactate dehydrogenase (LDH) have prognostic value in patients with metastatic melanoma and have recently been shown to predict and detect early response to immune checkpoint inhibition in metastatic melanoma [7,26]. Here we correlated the plasma ctDNA with serum LDH acquired contemporaneously.

**Figure 5:**
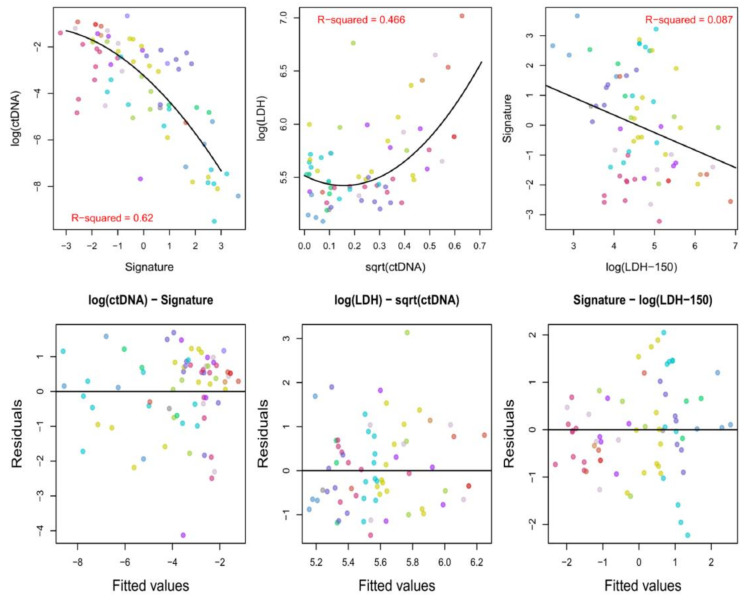
The interrelationship between log(ctDNA*maf*), serum LDH, and the radiomics signature in Equation (1). The three upper plots show the relationships between the radiomics signature and measured ctDNA*maf* (**upper left**); ctDNA*maf* and the measured LDH concentration (in UL^−1^, **upper middle**); LDH concentration and the radiomics signature (**upper right**), color-coded by patient. The variables are transformed in a way that yields the best fitting model in each case. The solid lines show the estimated mean values of the outcomes of interest at the population level as a function of their predictors, as fitted by means of random intercept mixed models. The three lower plots show the standardized residuals on the *y*-axes vs. the fitted values from the random intercept models used to assess the level of association between ctDNA*maf*, LDH concentrations and the PCA-based signature, color-coded by patient. The upper plots show a strong relationship between the radiomics signature and ctDNA*maf*, and also between LDH and ctDNA*maf*, but importantly there is no strong relationship between the radiomics signature and LDH: although the radiomics signature and LDH are both associated with ctDNA*maf* they are complementary. Note that ‘log’ denotes the natural logarithm.

The LDH local upper limit of normal (ULN) was 246 U/L. The relationship between the three variables—log(ctDNA*maf*), serum LDH, and the radiomics signature in Equation (1)—was assessed by means of random intercept mixed models, which are an extension of linear models to participant-dependent data. Model checks suggested a good fit of the assumed models to the data (Figure 5). As expected, the radiomics signature was associated with ctDNA*maf* levels in these patients (marginal R^2^ = 0.620 with a quadratic effect and 0.605 without) confirming the correctness of its derivation; a quadratic relationship best fitted the data. Measured LDH levels were also associated with ctDNA (marginal R^2^ = 0.466) and again a quadratic relationship was found to best fit the data. Importantly, the radiomics signature and LDH levels were not strongly related (marginal R^2^ = 0.087) suggesting they were nearly independent predictors of ctDNA*maf*.

## 3. Discussion

This study explored the interrelationship between two emerging diagnostic technologies in oncology, both of which offer the potential to make an impact on routine clinical practice: plasma ctDNA measurements as a very sensitive and specific tool for detection of tumor mutations, and radiomics as a method to quantitatively measure intratumoral and intertumoral spatial heterogeneity on routine clinical imaging such as CT. Patients with metastatic melanoma were chosen for the study given the high mutational burden of the cancer, the spatial and temporal variation in response to standard of care therapy seen in this patient group, and the heterogeneous imaging features of the disease, both in terms of the wide range of sites that it metastasizes to, as well as the variation of imaging features seen within and between tumors [27].

Our results show highly significant correlations between several radiomic features and the overall ctDNA*maf*. Further probing of this finding shows that much of this effect is due to the underlying correlation between ctDNA*maf* and lesion volume, an important consideration for future work in this field. We therefore investigated the added effect of radiomic features in predicting ctDNA*maf* controlling for lesion volume, and showed using a range of statistical approaches that a combination of radiomic features, in the form of a signature derived from PCA, had a significant association with ctDNA*maf* levels. The robustness of this finding was supported by several differing statistical techniques that yielded similar results.

The derived radiomics signature that best predicted ctDNA*maf* was a weighted sum of several individual imaging features. The biological correlates of most radiomic features and their significance on a tissue level is poorly understood, although there have been previous attempts to ascribe histological meaning to these values [28,29]. The simplest radiomic feature is the standard deviation of the histogram derived from the gray levels of voxels within the tumor, which reflects overall heterogeneity within the tumor voxels. Importantly the standard deviation made a significant contribution to the derived radiomics signature in this study. The more heterogeneous a tumor, the more likely it is to contain areas of proliferation and cell death, both of which will contribute to the overall ctDNA levels. The other radiomic features in the derived signature represent biological heterogeneity within the tumor and reflect the spatial relationship between tumor voxels using mathematical approaches which are more conceptually complex. No biopsy samples were available from the lesions chosen for study and it is therefore difficult to extrapolate the features in these samples to the CT imaging in this study. Important work is required in the future to correlate radiomic features and habitats with tissue from image-guided biopsies. By understanding the biological correlate of these textural features, radiomic analyses may complement invasive biopsies, particularly in the context of longitudinal tracking of tumor progression where multiple biopsies are not practical.

The biological alterations that lead to the release of ctDNA, such as tumor proliferation and necrosis, are nonspecific and final common pathways for most treatment regimes. Moreover, the radiomic features of heterogeneity or texture on CT detect morphological and functional tumor changes which are also relatively nonspecific. Any drug-specific effect will also be influenced by disease stage and whether they are used as first line or second line agents. Our hypothesis is therefore that the *correlation* between ctDNA and radiomics should be independent of the drug used and this has been borne out by the results we have presented here. For example, in our study ctDNA*maf* shows a significant increasing trend with time from the start of treatment (see Figure S9) which can be explained by ultimate disease progression irrespective of treatment. This is confirmed by the significant association of ctDNA*maf* levels with RECIST measures of treatment response (see Figure S10). We conclude from our main results that the radiomic signature also follows a similar trend since its overall prediction of ctDNA*maf* is statistically significant when taking into account readings at all time-points. Again, although we did detect a significant difference in the changes in ctDNA*maf* between patients undergoing BRAF inhibition and immunotherapy in the cohort studied here (see Figure S11), our results show that the radiomic feature signature reflects the treatment type in a similar way.

This study also explored the interrelationship between ctDNA, radiomic features, and LDH concentrations. LDH is an established, independent prognostic factor for disease survival [30,31] and has been shown to be an early biomarker for predicting treatment response in metastatic melanoma [7,26]. ctDNA levels have been shown to associate with blood LDH measurements in metastatic melanoma which is likely to be explained by the release of both intracellular nucleic acids and LDH as a consequence of tumor cell death [7]. Here, we also confirmed that measured LDH levels were associated with ctDNA levels. However, we found that the radiomics signature and LDH levels were not strongly related, suggesting they were nearly independent predictors of ctDNA*maf*. Although the radiomics signature and LDH measures are both predictive of ctDNA*maf* to some extent, our findings suggest that there are different factors governing each association and together they may be complementary. A possible explanation is that overall ctDNA*maf* is a measure of both proliferation and cell death, whereas LDH may largely probe the latter, and different radiomic features may probe either or both processes to differing degrees.

CT is the standard tool for measuring response to systemic therapy in the majority of metastatic cancers. However, recently there have been studies showing the potential role for ctDNA in predicting response to therapy [3,11,12,32]. Alterations in ctDNA have been reported to provide earlier markers of response to therapy than morphological changes in tumor size [33,34], and if individual mutations are unique to an individual metastasis, it may be possible to track the progress of individual metastases over time, analogous to the lesion-specific information provided by noninvasively imaging of a metastasis during treatment [4,35]. A recent study in metastatic melanoma reported that ctDNA levels at baseline and early follow-up can predict disease progression in patients treated with checkpoint inhibitors [36]. A further study showed that baseline ctDNA detection was associated with poor prognosis in metastatic *BRAF* or *NRAS*-mutated melanoma patients [37]. Similarly, the baseline mutant allele fraction and total level of ctDNA has been shown to be correlated with tumor burden in *BRAF*-mutated melanoma patients treated with combinational therapy including BRAF inhibitors [38].

However, many tumors including melanoma can demonstrate a mixed response to therapy and important aspects of intertumoral heterogeneity may be lost within the signal from the total tumor burden, if the focus is solely on the overall ctDNA*maf* measurements. Examples of this intertumoral variation between lesions can be seen in this study in Figure 3. Therefore, combining the sensitivity of ctDNA with metrics of tumor heterogeneity on imaging, may capture important and unique clinical information. Used together, these techniques could offer highly complementary information that could be used as part of integrative cancer care to better predict and detect response to cancer treatments [39,40]. 

This study has some limitations. Given the small study population and the novel and exploratory nature of this investigation, it was not possible to validate the findings on an independent dataset. The acquisition of a larger data set for further validation is being planned as future work and is consequently not part of the present study. In the meantime, we used several independent statistical methods which together support the robustness of our conclusions. In order to test the results, we undertook extensive Monte-Carlo simulations. By generating simulated data and comparing the results from this data with those from actual measurements it was possible to demonstrate the robustness of the statistics [41]. Another important factor to be considered in the field of radiomics is the consistency of the image acquisition parameters to enable repeatability and reproducibility of the textural features generated within and between patients [42,43,44]. These images were acquired as part of standard of care imaging undertaken in parallel with this prospective clinical study. To maintain quality control and minimize bias, the images analyzed were acquired at a single site using CT scanners from a single vendor and the radiomic analysis was performed on slices reconstructed into 5 mm in all patients. Not all of the blood samples were precisely contemporaneous with the CT imaging. This led to a median time difference from plasma sampling to imaging of 10 days and ctDNA measurements were interpolated from neighbor observations to correct for any difference in timing between the two. CT imaging was undertaken for the thorax, abdomen, and pelvis: any lesions outside the imaging field of view, particularly brain metastases, were therefore not included in the radiomic analysis. However, current evidence suggests that brain metastases do not significantly contribute to plasma ctDNA levels [45,46]. In addition, since there is no meaningful way of combining radiomics features from two or more separate volumes, we restricted the analysis to the largest lesion only as it has been established that ctDNA measurements are dependent on tumor volume. The patients studied were undergoing different systemic therapies and as such might be expected to follow differing disease progression pathways and for this reason, we did not draw conclusions about treatment response or survival.

The purpose of this study was to investigate the associations between ctDNA concentrations and CT imaging in metastatic melanoma at multiple individual time-points before, during, and after treatment. The presence of ctDNA in the plasma is secondary to nonspecific cellular processes which most therapeutics converge towards as part of the downstream consequences of their primary molecular mode of action; the correlations between ctDNA and CT demonstrated here are independent of therapy and show the potential future applications of this approach for generic response monitoring across different tumor subtypes and therapeutic interventions.

## 4. Materials and Methods

### 4.1. Patient Sample

This was a prospective study approved by the local institutional review board and research ethics committee (11/NE/0312) and managed within the Cambridge Clinical Trials Unit. Patients were recruited to the MelResist study which evaluated response and resistance biomarkers in metastatic melanoma patients undergoing systemic therapy. Written informed consent was obtained from all patients before enrolment into the study.

CT imaging, blood plasma ctDNA samples and LDH measurements were concurrently obtained from 15 patients (10 males, 5 females; median age 62, range 33–72). Each of the patients had CT examinations at regular intervals (approximately 2-monthly; median interval and inter-quartile range = 60 (55, 82)) days throughout their treatment regime as part of standard of care management and the correlation between imaging and ctDNA was performed retrospectively (pretreatment, concurrent with treatment and post-treatment). Table 1 presents patient information in more detail.

### 4.2. Image Acquisition and Analysis

CT imaging was performed at a single site using scanners from a single vendor (Siemens, Erlangen, Germany) but on differing individual machines. All the radiomic analyses were performed on images with a reconstructed slice thickness of 5 mm. The acquisition parameters were as follows: reconstruction kernel B31s or B41s; tube voltage (median: 130; and range: 80, 140; kV); exposure (mean ± s.d.: 95 ± 62 mA s). All the analyzed CT scans were encoded in the Digital Imaging and Communications in Medicine (DICOM) format with 16 bit-depth.

All lesions were identified from radiology reports and then outlined by an experienced observer (A.G.) and a radiologist (J.Z., R.W., F.Z.) with more than 5 years of experience. The regions of interest (ROI) were subsequently reviewed and if necessary edited by a radiologist (F.G.) with 10 years of experience as an attending radiologist. Outlining was performed using custom software written in MATLAB version 2018a (The Mathworks, Natick, MA, USA). Lesions with a volume consistently less than 1 cm^3^ were not included in the analysis.

The ROIs were then converted using custom software (written in MATLAB) to the Neuroimaging Informatics Technology Initiative (NIfTI) format [47], suitable for importing into the LIFEx software package for textural analysis. Scripts to automate LIFEx processing were generated by custom software written in MATLAB. Each individual lesion was analyzed as a separate volume.

LIFEx was configured to extract textural parameters after re-binning the lesion images into 128 gray levels and an absolute range of CT numbers from −400 to 400 Hounsfield units (HU). In a second software run, gray level zone length features were extracted after binning the same range of CT numbers into 32 gray levels following the recommendations of the image biomarker standardization initiative (IBSI) for this type of feature [48]. Spatial resampling to 1 mm isotropic voxels was preapplied to standardize for pixel size and slice thickness variations. A range of output variables totaling 39 image features was produced comprising histogram measures, shape features, gray level co-occurrence (GLCM) matrix features [49,50], neighborhood gray level difference matrix (NGLDM) features [51], gray level run length matrix (GLRLM) features [52], and gray level zone length matrix (GLZLM) features [53]. A histogram of gray levels for each lesion was generated at each imaging time-point: these were inspected to confirm that the histogram range (−400, 400) HU encompassed the range of gray-values within the lesion.

Where more than one lesion was identified in an individual patient (i.e., in 12 of the 15 patients), textural measures were taken from the largest lesion. The largest lesion was selected as that with the greatest volume at any time-point and subsequently this individual lesion was tracked across all time-points to provide textural metrics.

Image features were generated for each patient visit for routine CT imaging: there was a total of 70 such visits across all patients yielding a results table of 70 observations and 39 variables, representing the full range of applicable texture features reported by LIFEx.

Feature maps were calculated for one example lesion using the PyRadiomics (version 3.0) [54] package in Python and custom software also written in Python (version 3.7.6, Python Software Foundation, Wilmington, DE, USA). The kernel (i.e., sliding window) radius for the mapping was set to 1 pixel and all other parameters matched those used in LIFEx.

### 4.3. ctDNA Quantification

Plasma sample processing, library preparation, and shallow whole genome sequencing (sWGS) of plasma samples were performed as previously described [34]. Peripheral blood samples were collected at each clinic visit in S-Monovette 9 mL EDTA tubes. To isolate plasma, whole blood samples were centrifuged at 1600× *g* for 10 min within an hour of the blood draw, followed by an additional centrifugation of plasma supernatant 20,000× *g* for 10 min. Samples were stored at −80 °C. Plasma samples were extracted using a QIAamp protocol using a QIAsymphony (Qiagen, Hilden, Germany). Library preparation was performed with the Rubicon ThruPLEX Plasma-Seq kit, using between 7 and 15 PCR cycles, as recommended by the manufacturer (Rubicon Genomics, Ann Arbor, MI, USA).

Libraries were sequenced on a HiSeq 4000 (Illumina, San Diego, CA, USA), and copy number analysis performed using ichorCNA (Broad Institute of MIT and Harvard, Cambridge, MA, USA) using the default settings without a matched panel of normals [55]. Tumor fractions from ichorCNA analysis of plasma cell-free DNA were used as a measure for ctDNA level, as described previously [55].

For samples with tumor fractions determined by sWGS and ichorCNA <3% (i.e., below the limit of detection of sWGS [55]), TAm-Seq was performed as previously described to gain extra sensitivity [5]. Targeted sequencing assays for TAm-Seq were developed, as part of personalized sequencing panels which included amplicons, targeting the following mutation loci: BRAF V600 (chr7:140453136) (C > T) or NRAS Q61 (chr1:115256530) (G > T) [56]. For TAm-Seq, mutant allele fraction was determined for each locus in each sample.

ctDNA*maf* levels, as determined by either targeted sequencing or sWGS, were compared against imaging data at matched time points or interpolated (using linear interpolation) between neighboring observations when imaging and blood sampling time-points did not match.

### 4.4. Statistical Analysis Methods

Statistical analysis was conducted with the R statistical program (version 4.0.0) [57] and its packages lmerTest (3.1-2, https://cran.r-project.org/web/packages/lmerTest/index.html), MuMIn (1.43.17, https://cran.r-project.org/web/packages/MuMIn/index.html), robustlmm (2.3, https://cran.r-project.org/web/packages/robustlmm/index.html), and lmmlasso (0.1-2, https://cran.r-project.org/web/packages/lmmlasso/index.html) in three phases summarized in Figure 2.

#### 4.4.1. Descriptive Analyses and Data Transformation

Scatter plots were generated allowing visualization of the relationship between each feature on a given scale and log(ctDNA*maf*). They allowed for a visual assessment of the suitability of the bivariate normal assumption (a conditional normal assumption being required by later analyses), detection of the presence of outliers and within-participant dependence. Where necessary, feature variables were transformed (e.g., to log(*f*) or log(*a* + *f*) or log(log(*x*) + *a*), where ‘*f*’ represents the feature in question and ‘a’ a constant) in order to linearize their relationship with log(ctDNA*maf*), decrease heteroscedasticity and minimize the effect of outliers.

#### 4.4.2. Analysis without Controlling for Lesion Volume

The analysis of the relationship between the radiomic features of interest and log(ctDNA*maf*) without correcting for lesion volume was performed in two ways.

Firstly, feature-by-feature analysis was performed by means of a random intercept mixed linear model (MLM) with log(ctDNA*maf*) as the response. The feature of interest on the chosen scale was the predictor and patients were added as a random effect to take the within-patient dependence into account. Parameter estimates were obtained by means of the restricted maximum likelihood estimator (REML) [58]. Wald *t*-test statistics corresponding to each feature were compared to the statistics of a robust estimator [59], the latter being less sensitive to model deviations. This served as a sensitivity analysis for outliers. The Benjamini–Hochberg false discovery rate (FDR) multiplicity correction was used when assessing statistical significance, aiming to keep the global FDR at the 5% level [60]. The ability of the multiplicity correction to distinguish genuine and nongenuine relationships between features and ctDNA*maf* was assessed by considering an additional set of 100 independent random predictors. These were generated from a Gaussian distribution both with and without patient dependence, assuming a similar intracluster correlation as observed in the real data. Marginal R-squared, representing the variance of log(ctDNA*maf*) explained by the fixed predictors when ignoring patient effects [61], was used to assess the model performance.

Secondly, global analysis was performed by means of two model selection procedures suitable for data showing within-cluster dependence. These considered log(ctDNA*maf*) as the response and the set of 39 features on the transformed scale as predictors. The first procedure used a least absolute shrinkage and selection operator (LASSO) model for dependent data [62] and considered standardized predictors, as required by such techniques [63]. The regularization parameter value (λ) was selected by iteratively increasing its value until the first random predictor (among a set of random predictors the same size as the set of real predictors, i.e., 39) was included in the model. The second procedure used a stepwise forward/backward model selection maximizing the marginal R-square of the MLM [61]. In a similar fashion as to that employed in the LASSO approach, random predictors were used to govern when the iteration steps should be halted.

As sensitivity analyses, the results of these two procedures were compared to the ones obtained when removing observations considered as outliers. The outliers in turn were identified by estimating the covariance matrix of the full dataset, i.e., log(ctDNA*maf*) and the 39 features of interest, by means of a robust S-estimator [64].

#### 4.4.3. Analysis Controlling for Lesion Volume

Scatter plots were generated to visualize the relationship between each feature on the transformed scale and the lesion volume. The level of association was measured by means of the Spearman ρ statistic. The same feature-by-feature models, estimators, and multiplicity correction were employed as described above, but with lesion volume as an additional fixed predictor. Likelihood ratio tests (LRT), comparing models with the radiomic feature as an additional predictor (full model) to the model with lesion volume as the only predictor (restricted model), and Wald *t*-tests were used to assess the additional effect of the radiomic feature to predict ctDNA mutant allele fraction over volume alone.

Secondly, the radiomic features yielding the five smallest *p*-values of the LRT in the previous analysis step were used to jointly predict log(ctDNA*maf*), either directly or through a signature defined as the first dimension of a PCA fitted on the standardized predictors.

Simulated data were used to assess the performance of these two models on the real data-set. A total of 2500 new data-sets of 38 random features (volume was excluded from the original list of 39) were generated with the same correlation structure as that observed with the real features, including the correlation with lesion volume, but with a correlation of zero with log(ctDNA*maf*). This was performed both with and without patient dependence (assuming a similar intracluster correlation as observed in the real data).

A comparison was made between marginal R-squared and LRT statistics (calculated from the selection of the five best features based on LRT *p*-values from feature by feature models and the derived PCA) both from the real data-set and the set of 2500 simulated data-sets.

The distribution of the marginal R-squared and LRT statistics based on random sets of features correspond to what would be observed if the radiomic features had no predictive power “over and above” lesion volume, while preserving the observed relationship between lesion volume and log(ctDNA*maf*).

#### 4.4.4. Analysis of the Associations between ctDNA, LDH Levels, and the Derived Radiomics Signature

ctDNA*maf*, LDH levels, and the PCA-based signature, derived as described in the previous section, were transformed in order to linearize their relationship with each other, decrease heteroscedasticity, and minimize the effect of outliers. Their relationships after transformation were visualized by means of scatter plots, with observations color-coded by patient.

Random intercept mixed models with the patient as a random effect were then used to model their relationships while accounting for the within-patient dependence. The quality of the fit and type of model (linear vs. quadratic) were derived by means of observation of the relationship between the standardized residuals and fitted values. The strength of the relationship was assessed by means of marginal R-squared [61].

## 5. Conclusions

In conclusion, this study investigated the interrelationship between heterogeneity on imaging using radiomics and plasma ctDNA levels in metastatic melanoma patients. ctDNA mutant allele fraction significantly correlated with the overall tumor volume. However, a radiomics signature derived by principal component analysis as a measure of tumor heterogeneity predicted ctDNA levels independently of tumor volume and serum LDH, and therefore provided additional complementary information. The study suggests the feasibility of combining radiomics and liquid biomarkers to monitor changes in tumor heterogeneity in patients during their treatment course. Radiomic features and ctDNA*maf* could be complementary clinical tools, and by combining both imaging and liquid biopsy approaches, tumors can be more deeply phenotyped. Together, these techniques may provide robust measures for monitoring tumors in future clinical practice.

## Figures and Tables

**Table 1:** Clinical characteristics of study cohort.

Patient ID	Gender	Lesion Analyzed	Intra-Axial Brain Lesions ^1^	AJCC Stage	BRAF Mutation Status	Baseline Serum LDH (UL)	Treatment	CT Visit Number	RECIST 1.1 Response Assessment	Target Lesion: Descriptive Volume Changes ^2^	Lesion Volume at Baseline (mL)	Target Lesion: Fractional Volume at Baseline (%) ^3^	Number of Lesions (>1 cm^3^)
1	M	Left axillary lymph node metastasis	N	IV	+	-	Vemurafenib	i	Progressive Disease	Baseline	45	58	2
								ii	Partial Response	Large decrease			
2	M	Para-aortic lymph node metastasis	N	IV	+	261	Vemurafenib	i	Partial Response	Baseline	65	93	4
								ii	Partial Response	Large decrease			
								iii	Partial Response	Large decrease			
								iv	Partial Response	Large decrease			
								v	Partial Response	Large decrease			
								vi	Progressive Disease	Small increase			
								vii	Stable Disease	Large increase (borderline)			
								viii	Progressive Disease	Large increase			
								ix	Progressive Disease	Large increase			
3	M	Axillary lymph node metastasis left	Y	IV	+	252	Vemurafenib	i	Progressive Disease	Baseline	3	63	7
								ii	Partial Response	Large decrease			
								iii	Stable Disease	Small decrease			
								iv	Stable Disease	Small decrease			
								v	Stable Disease	Small decrease			
							Ipilimumab	vi	Progressive Disease	Large increase			
								vii	Progressive Disease	Large increase			
							Pazopanib	viii	Progressive Disease	Large increase			
								ix	Stable Disease	Small increase			
							Dabrafenib/Trametinib	x	Progressive Disease	Small increase			
								xi	Progressive Disease	Large increase			
4	F	Iliac lymph node metastasis right	N	IV	+	246	Vemurafenib	i	Stable Disease	Baseline	7	99	2
								ii	Stable Disease	Small decrease			
								iii	Stable Disease	Small decrease			
								iv	Stable Disease	Small decrease			
								v	Stable Disease	Small decrease			
								vi	Stable Disease	Large increase			
								vii	Stable Disease	Large increase			
								viii	Progressive Disease	Large increase			
								ix	Progressive Disease	Large increase			
							pan-RAF inhibitor	x	Stable Disease	Small decrease			
								xi	Stable Disease	Small increase			
								xii	Progressive Disease	Large increase			
5	M	Omental metastasis	U	IV	+	258	Vemurafenib	i	Partial Response	Baseline	6	100	6
								ii	Progressive Disease	Large increase			
							Ipilimumab	iii	Progressive Disease	Small increase			
6	M	Omental metastasis	N	IV	+	773	Dabrafenib/Trametinib	i	Progressive Disease	Baseline	461	44	6
								ii	Partial Response	Large decrease			
								iii	Partial Response	Large decrease			
								iv	Progressive Disease	Large decrease (borderline)			
								v	Progressive Disease	Large increase			
7	M	Right axillary lymph node	Y	IV	+	699	Vemurafenib	i	Progressive Disease	Baseline	296	95	4
8	F	Femoral, subcutaneous metastasis right	Y	IV	+	175	Vemurafenib	i	Progressive Disease	Baseline	1	100	1
								ii	Stable Disease	Small decrease			
								iii	Stable Disease	Small increase			
								iv	Stable Disease	Small decrease			
								v	Progressive Disease	Large increase			
9	F	Dorsal subcutaneous metastasis abutting the iliac crest	N	IV	+	201	Dabrafenib	i	n/a	Baseline	2	93	4
								ii	n/a	Large increase			
								iii	n/a	Large increase			
10	M	Right external iliac lymph node metastasis	Y	IV	+	254	Dabrafenib/Trametinib	i	Partial Response	Baseline	9	75	2
								ii	Partial Response	Small decrease			
								iii	Partial Response	Small decrease			
								iv	Partial Response	Large decrease			
								v	Stable Disease	Small increase			
								vi	Stable Disease	Large increase (borderline)			
								vii	Progressive Disease	Large increase			
11	M	Left supraclavicular lymph node metastasis	Y	IV	+	169	Vemurafenib	i	Stable Disease	Baseline	9	100	1
12	F	Splenic lymph node metastasis	U	IV	+	233	No treatment	i	Progressive Disease	Baseline	58	56	2
13	M	Left axillary lymph node metastasis	U	IV	+	492	Vemurafenib	i	Progressive Disease	Baseline	136	100	1
14	M	Left iliac lymph node metastasis	Y	IV	+	351	Dabrafenib/Trametinib	i	Progressive Disease	Baseline	11	31	5
								ii	Partial Response	Small increase			
								iii	Progressive Disease	Small increase			
								iv	Progressive Disease	Large increase			
							Ipilimumab	v	Progressive Disease	Small increase			
15	F	Left inguinal lymph node metastasis	Y	IV	−	392	pan-RAF inhibitor	i	Progressive Disease	Baseline	63	78	4
								ii	Progressive Disease	Small increase			
							Ipilimumab	iii	Stable Disease	Small decrease			
							Pembrolizumab	iv	Stable Disease	Small decrease			
								v	Stable Disease	Small decrease			
								vi	Stable Disease	Small increase			

^1^ Key: Y—Yes; N—No; U—Unknown. ^2^ Volume changes in the target lesion were categorized as follows: large increase is >73% increase; large decrease is >66% decrease; small increase is <73% increase; small decrease is <66% decrease. These thresholds were calculated from RECIST 1.1 diameter thresholds, converting to expected volume changes: e.g., 0.73 = (1 + 0.2)^3^ − 1. ^3^ Fractional volume was calculated over the sum of volumes of lesions imaged on standard CT.

**Table 2:** Comparison of radiomic features to log(ctDNA*maf*) without controlling for lesion volume.

^1^ Feature	*t*-Value	*p*-Value	Adj. *p*-Value	^2^ Sig.	R^2^
GLNUz	8.263	1.11 × 10^-11^	1.54 × 10^-9^	***	0.517
GLNUr	7.531	7.49 × 10^-10^	2.80 × 10^-8^	***	0.496
Coarseness	−7.527	6.86 × 10^-10^	2.80 × 10^-8^	***	0.495
ZLNU	7.273	8.06 × 10^-10^	2.80 × 10^-8^	***	0.458
RLNU	7.327	1.43 × 10^-9^	3.98 × 10^-8^	***	0.477
Volume	7.295	1.79 × 10^-9^	4.16 × 10^-8^	***	0.476
Busyness	6.608	1.57 × 10^-8^	3.12 × 10^-7^	***	0.428
Contrast	−6.143	2.81 × 10^-7^	4.89 × 10^-6^	***	0.434
SRLGE	−5.973	3.75 × 10^-7^	5.80 × 10^-6^	***	0.411
LGRE	−5.673	7.99 × 10^-7^	1.11 × 10^-5^	***	0.376
StdDev	−5.137	2.59 × 10^-6^	3.27 × 10^-5^	***	0.295
Mean	5.218	4.24 × 10^-6^	4.92 × 10^-5^	***	0.331
ZP	−5.069	4.67 × 10^-6^	5.00 × 10^-5^	***	0.298
HGRE	5.075	6.81 × 10^-6^	6.76 × 10^-5^	***	0.321
Entropy_h	−4.850	8.19 × 10^-6^	7.59 × 10^-5^	***	0.285
LRHGE	4.703	3.04 × 10^-5^	2.65 × 10^-4^	***	0.306
Energy	4.494	3.30 × 10^-5^	2.70 × 10^-4^	***	0.262
Correlation	−3.676	4.80 × 10^-4^	3.51 × 10^-3^	**	0.191
SRHGE	3.699	4.75 × 10^-4^	3.51 × 10^-3^	**	0.192
LGZE	−3.740	5.23 × 10^-4^	3.64 × 10^-3^	**	0.217
Sphericity	−3.859	5.84 × 10^-4^	3.87 × 10^-3^	**	0.230
Kurtosis	3.586	6.84 × 10^-4^	4.32 × 10^-3^	**	0.198
LRE	3.522	1.04 × 10^-3^	6.26 × 10^-3^	**	0.194
SRE	−3.456	1.26 × 10^-3^	7.29 × 10^-3^	**	0.191
RP	−3.428	1.35 × 10^-3^	7.53 × 10^-3^	**	0.187
Uniformity	3.189	2.23 × 10^-3^	1.19 × 10^-2^	*	0.146
[random35]	2.975	4.09 × 10^-3^	2.11 × 10^-2^	*	0.095

Wald *t*-statistics, corresponding *p*-values with and without FDR multiplicity correction for the combined set of real and random features, and marginal R-squared estimates of feature by feature mixed linear models relating radiomic features to log(ctDNA*maf*). ^1^ Radiomic features: GLNUr/z—gray level nonuniformity (run length/zone length); ZLNU—zone length nonuniformity; RLNU—run-length nonuniformity; SRLGE—short-run low gray level emphasis; ZP—zone percentage; LGRE—low gray level run emphasis; HGRE—high gray level run emphasis; LRHGE—long-run high gray level emphasis; SRHGE—short-run high gray level emphasis; LGZE—low gray level zone emphasis; SRE—short-run emphasis; LRE—long run emphasis; RP—run percentage; ZP—zone percentage; Entropy_h—histogram entropy; SRHGE—short-run high gray level emphasis. See Table S5 for a key to the feature names. ^2^ *** *p* < 0.001, ** *p* < 0.01, * *p* < 0.05.

**Table 3:** Predictors (top 20) of ctDNA*maf* calculated from feature-by-feature linear mixed models with lesion volume included as a fixed effect.

Feature	*t*-Value	*p*-Value	Adjusted *p*-Value	R^2^
Correlation	−3.227	0.0020	0.1708	0.536
GLNUz	3.151	0.0025	0.1708	0.515
StdDev	−2.800	0.0067	0.2992	0.531
LGRE	−2.700	0.0105	0.2992	0.536
[random78]	−2.506	0.0149	0.2992	0.514
Coarseness	−2.512	0.0150	0.2992	0.515
[random45]	2.497	0.0152	0.2992	0.508
Mean	2.419	0.0206	0.3060	0.523
SRHGE	2.359	0.0230	0.3060	0.518
[random83]	2.322	0.0234	0.3060	0.506
LRLGE	−2.300	0.0259	0.3060	0.506
HGRE	2.308	0.0266	0.3060	0.520
[random77]	2.135	0.0367	0.3670	0.501
[random80]	−2.126	0.0372	0.3670	0.498
SRLGE	−2.131	0.0399	0.3672	0.519
GLNUr	2.034	0.0459	0.3736	0.509
[random91]	−2.034	0.0460	0.3736	0.496
[random79]	−2.005	0.0490	0.3758	0.502
[random35}	1.851	0.0687	0.4853	0.488
Entropy_h	−1.839	0.0703	0.4853	0.504

## Supplementary Materials

The following are available online at https://www.mdpi.com/2072-6694/12/12/3493/s1, Figure S1: Scatterplot showing concordance between shallow WGS ichorCNA tumor fraction and TAm-Seq ctDNA*maf* measurements, where samples were acquired on the same day, Figure S2: The relationship between each radiomic feature, after the scale transformation, [*x*-axes] and log(ctDNA*maf*) [*y*-axis], color-coded by patient, Figure S3: Marginal R-squared (*y*-axis) plotted with multiplicity adjusted *p*-values (*x*-axis), colour-coded by feature type, when explaining log(ctDNA*maf*) without controlling for lesion volume, Figure S4: Matrix scatter plot of the radiomic features that showed a significant relationship with ctDNA*maf*, with observations colour coded by patient, Figure S5: Comparison of the test statistics of the 39 features of interest when fitting feature by feature by means of REML (*x*-axis) and a robust estimator (*y*-axis), color coded by multiplicity adjusted p-values, Figure S6: The relationship between each feature (*x*-axes) and lesion volume (*y*-axis), color-coded by patient, Figure S7: Marginal R-squared (*y*-axis) plotted against multiplicity adjusted *p*-values (*x*-axis), color-coded by feature type, when explaining log(ctDNA*maf*) in addition to the effect of lesion volume, Figure S8: Sample radiomic feature maps within a large tumor (from patient 3), Figure S9: ctDNA with time from treatment start. ctDNA*maf* (on the log scale, *y*-axis) as a function of time from start of treatment as a dichotomous variable with observation color coded by patients, Figure S10: ctDNA*maf* on a log scale compared to RECIST treatment response, Figure S11: ctDNA*maf* (on the log scale, *y*-axis) as a function of treatment drug type, where observations are color coded by patient, Table S1: ctDNA assays listed by patient blood sample time-points, Table S2: Interpolated ctDNA*maf* readings listed by patient CT imaging visit., Table S3: Statistics from feature-by-feature mixed linear models predicting ctDNA*maf* without controlling for lesion volume., Table S4: Statistics from feature-by-feature mixed linear models predicting ctDNA*maf* with lesion volume included as a fixed effect, Table S5: Key to radiomics feature nomenclature.

## References

[1] Schwarzenbach H., Hoon D.S., Pantel K. (2011). Cell-free nucleic acids as biomarkers in cancer patients. Nat. Rev. Cancer.

[2] Diehl F., Schmidt K., Choti M.A., Romans K., Goodman S., Li M., Thornton K., Agrawal N., Sokoll L., Szabo S.A. (2008). Circulating mutant DNA to assess tumor dynamics. Nat. Med..

[3] Dawson S.J., Tsui D.W., Murtaza M., Biggs H., Rueda O.M., Chin S.F., Dunning M.J., Gale D., Forshew T., Mahler-Araujo B. (2013). Analysis of circulating tumor DNA to monitor metastatic breast cancer. N. Engl. J. Med..

[4] Murtaza M., Dawson S.J., Pogrebniak K., Rueda O.M., Provenzano E., Grant J., Chin S.F., Tsui D.W.Y., Marass F., Gale D. (2015). Multifocal clonal evolution characterized using circulating tumour DNA in a case of metastatic breast cancer. Nat. Commun..

[5] Forshew T., Murtaza M., Parkinson C., Gale D., Tsui D.W.Y., Kaper F., Dawson S.J., Piskorz A.M., Jimenez-Linan M., Bentley D. (2012). Noninvasive Identification and Monitoring of Cancer Mutations by Targeted Deep Sequencing of Plasma DNA. Sci. Transl. Med..

[6] Wan J.C.M., Massie C., Garcia-Corbacho J., Mouliere F., Brenton J.D., Caldas C., Pacey S., Baird R., Rosenfeld N. (2017). Liquid biopsies come of age: Towards implementation of circulating tumour DNA. Nat. Rev. Cancer.

[7] Lee J.H., Long G.V., Menzies A.M., Lo S., Guminski A., Whitbourne K., Peranec M., Scolyer R., Kefford R.F., Rizos H. (2018). Association Between Circulating Tumor DNA and Pseudoprogression in Patients With Metastatic Melanoma Treated With Anti-Programmed Cell Death 1 Antibodies. JAMA Oncol..

[8] Lu H.N., Arshad M., Thornton A., Avesani G., Cunnea P., Curry E., Kanavati F., Liang J., Nixon K., Williams S.T. (2019). A mathematical-descriptor of tumor-mesoscopic-structure from computed-tomography images annotates prognostic- and molecular-phenotypes of epithelial ovarian cancer. Nat. Commun..

[9] Abbosh C., Birkbak N.J., Wilson G.A., Jamal-Hanjani M., Constantin T., Salari R., Le Quesne J., Moore D.A., Veeriah S., Rosenthal R. (2017). Phylogenetic ctDNA analysis depicts early-stage lung cancer evolution. Nature.

[10] Parkinson C.A., Gale D., Piskorz A.M., Biggs H., Hodgkin C., Addley H., Freeman S., Moyle P., Sala E., Sayal K. (2016). Exploratory Analysis of TP53 Mutations in Circulating Tumour DNA as Biomarkers of Treatment Response for Patients with Relapsed High-Grade Serous Ovarian Carcinoma: A Retrospective Study. PLoS Med..

[11] Osumi H., Shinozaki E., Yamaguchi K., Zembutsu H. (2019). Early change in circulating tumor DNA as a potential predictor of response to chemotherapy in patients with metastatic colorectal cancer. Sci. Rep..

[12] Chaudhuri A.A., Binkley M.S., Osmundson E.C., Alizadeh A.A., Diehn M. (2015). Predicting Radiotherapy Responses and Treatment Outcomes Through Analysis of Circulating Tumor DNA. Semin. Radiat. Oncol..

[13] Eisenhauer E.A., Therasse P., Bogaerts J., Schwartz L.H., Sargent D., Ford R., Dancey J., Arbuck S., Gwyther S., Mooney M. (2009). New response evaluation criteria in solid tumours: Revised RECIST guideline (version 1.1). Eur. J. Cancer.

[14] Lambin P., Rios-Velazquez E., Leijenaar R., Carvalho S., Van Stiphout R.G., Granton P., Zegers C.M., Gillies R., Boellard R., Dekker A. (2012). Radiomics: Extracting more information from medical images using advanced feature analysis. Eur. J. Cancer.

[15] Jamal-Hanjani M., Quezada S.A., Larkin J., Swanton C. (2015). Translational implications of tumor heterogeneity. Clin. Cancer Res..

[16] Larue R.T., Defraene G., De Ruysscher D., Lambin P., Van Elmpt W. (2017). Quantitative radiomics studies for tissue characterization: A review of technology and methodological procedures. Br. J. Radiol..

[17] Gillies R.J., Kinahan P.E., Hricak H. (2016). Radiomics: Images Are More than Pictures, They Are Data. Radiology.

[18] Aerts H.J., Velazquez E.R., Leijenaar R.T., Parmar C., Grossmann P., Carvalho S., Bussink J., Monshouwer R., Haibe-Kains B., Rietveld D. (2014). Decoding tumour phenotype by noninvasive imaging using a quantitative radiomics approach. Nat. Commun..

[19] Sala E., Mema E., Himoto Y., Veeraraghavan H., Brenton J.D., Snyder A., Weigelt B., Vargas H.A. (2017). Unravelling tumour heterogeneity using next-generation imaging: Radiomics, radiogenomics, and habitat imaging. Clin. Radiol..

[20] Goldberg S.B., Patel A.A. (2018). Monitoring immunotherapy outcomes with circulating tumor DNA. Immunotherapy.

[21] Thierry A.R., Mouliere F., Gongora C., Ollier J., Robert B., Ychou M., Del Rio M., Molina F. (2010). Origin and quantification of circulating DNA in mice with human colorectal cancer xenografts. Nucleic Acids Res..

[22] Kamat A.A., Bischoff F.Z., Dang D., Baldwin M.F., Han L.Y., Lin Y.G., Merritt W.M., Landen C.N., Lu C., Gershenson D.M. (2006). Circulating cell-free DNA: A novel biomarker for response to therapy in ovarian carcinoma. Cancer Biol. Ther..

[23] Forschner A., Battke F., Hadaschik D., Schulze M., Weissgraeber S., Han C.T., Kopp M., Frick M., Klumpp B., Tietze N. (2019). Tumor mutation burden and circulating tumor DNA in combined CTLA-4 and PD-1 antibody therapy in metastatic melanoma—Results of a prospective biomarker study. J. Immunother. Cancer.

[24] Lin L.I. (1989). A concordance correlation coefficient to evaluate reproducibility. Biometrics.

[25] Nioche C., Orlhac F., Boughdad S., Reuze S., Goya-Outi J., Robert C., Pellot-Barakat C., Soussan M., Frouin F., Buvat I. (2018). LIFEx: A Freeware for Radiomic Feature Calculation in Multimodality Imaging to Accelerate Advances in the Characterization of Tumor Heterogeneity. Cancer Res..

[26] Diem S., Kasenda B., Spain L., Martin-Liberal J., Marconcini R., Gore M., Larkin J. (2016). Serum lactate dehydrogenase as an early marker for outcome in patients treated with anti-PD-1 therapy in metastatic melanoma. Br. J. Cancer.

[27] Yi X., Ma J., Guan Y., Chen R., Yang L., Xia X. (2017). The feasibility of using mutation detection in ctDNA to assess tumor dynamics. Int. J. Cancer.

[28] Yip S.S., Aerts H.J. (2016). Applications and limitations of radiomics. Phys. Med. Biol..

[29] Parekh V., Jacobs M.A. (2016). Radiomics: A new application from established techniques. Expert Rev. Precis. Med. Drug Dev..

[30] Eton O., Legha S.S., Moon T.E., Buzaid A.C., Papadopoulos N.E., Plager C., Burgess A.M., Bedikian A.Y., Ring S., Dong Q. (1998). Prognostic factors for survival of patients treated systemically for disseminated melanoma. J. Clin. Oncol..

[31] Weide B., Elsasser M., Buttner P., Pflugfelder A., Leiter U., Eigentler T.K., Bauer J., Witte M., Meier F., Garbe C. (2012). Serum markers lactate dehydrogenase and S100B predict independently disease outcome in melanoma patients with distant metastasis. Br. J. Cancer.

[32] Siravegna G., Marsoni S., Siena S., Bardelli A. (2017). Integrating liquid biopsies into the management of cancer. Nat. Rev. Clin. Oncol..

[33] Butler T.M., Spellman P.T., Gray J. (2017). Circulating-tumor DNA as an early detection and diagnostic tool. Curr. Opin. Genet. Dev..

[34] Wan J.C.M., Heider K., Gale D., Murphy S., Fisher E., Mouliere F., Ruiz-Valdepenas A., Santonja A., Morris J., Chandrananda D. (2020). ctDNA monitoring using patient-specific sequencing and integration of variant reads. Sci. Transl. Med..

[35] Durot C., Mule S., Soyer P., Marchal A., Grange F., Hoeffel C. (2019). Metastatic melanoma: Pretreatment contrast-enhanced CT texture parameters as predictive biomarkers of survival in patients treated with pembrolizumab. Eur. Radiol..

[36] Pedersen J.G., Madsen A.T., Gammelgaard K.R., Aggerholm-Pedersen N., Sorensen B.S., Ollegaard T.H., Jakobsen M.R. (2020). Inflammatory Cytokines and ctDNA Are Biomarkers for Progression in Advanced-Stage Melanoma Patients Receiving Checkpoint Inhibitors. Cancers.

[37] Herbreteau G., Vallee A., Knol A.C., Theoleyre S., Quereux G., Frenard C., Varey E., Hofman P., Khammari A., Dreno B. (2020). Circulating Tumour DNA Is an Independent Prognostic Biomarker for Survival in Metastatic BRAF or NRAS-Mutated Melanoma Patients. Cancers.

[38] Ahlborn L.B., Tuxen I.V., Mouliere F., Kinalis S., Schmidt A.Y., Rohrberg K.S., Santoni-Rugiu E., Nielsen F.C., Lassen U., Yde C.W. (2018). Circulating tumor DNA as a marker of treatment response in BRAF V600E mutated non-melanoma solid tumors. Oncotarget.

[39] Hesketh R.L., Zhu A.X., Oklu R. (2015). Radiomics and circulating tumor cells: Personalized care in hepatocellular carcinoma?. Diagn. Interv. Radiol..

[40] Neri E., Del Re M., Paiar F., Erba P., Cocuzza P., Regge D., Danesi R. (2018). Radiomics and liquid biopsy in oncology: The holons of systems medicine. Insights Imaging.

[41] Efron B. (1981). Nonparametric estimates of standard error: The jackknife, the bootstrap and other methods. Biometrika.

[42] Lu L., Liang Y., Schwartz L.H., Zhao B. (2019). Reliability of Radiomic Features Across Multiple Abdominal CT Image Acquisition Settings: A Pilot Study Using ACR CT Phantom. Tomography.

[43] Shafiq-Ul-Hassan M., Latifi K., Zhang G., Ullah G., Gillies R., Moros E. (2018). Voxel size and gray level normalization of CT radiomic features in lung cancer. Sci. Rep..

[44] Mackin D., Fave X., Zhang L., Fried D., Yang J., Taylor B., Rodriguez-Rivera E., Dodge C., Jones A.K., Court L. (2015). Measuring Computed Tomography Scanner Variability of Radiomics Features. Investig. Radiol..

[45] Bettegowda C., Sausen M., Leary R.J., Kinde I., Wang Y., Agrawal N., Bartlett B.R., Wang H., Luber B., Alani R.M. (2014). Detection of circulating tumor DNA in early- and late-stage human malignancies. Sci. Transl. Med..

[46] Lee J.H., Menzies A.M., Carlino M.S., McEvoy A.C., Sandhu S., Weppler A.M., Diefenbach R.J., Dawson S.J., Kefford R.F., Millward M.J. (2020). Longitudinal Monitoring of ctDNA in Patients with Melanoma and Brain Metastases Treated with Immune Checkpoint Inhibitors. Clin. Cancer Res..

[47] Cox R.W., Ashburner J., Breman H., Fissell K., Haselgrove C., Holmes C.J., Lancaster J.L., Rex D.E., Smith S.M., Woodward J.B. A (sort of) new image data format standard: NIfTI-1. Proceedings of the 10th Annual Meeting of the Organization for Human Brain Mapping.

[48] Zwanenburg A., Vallieres M., Abdalah M.A., Aerts H., Andrearczyk V., Apte A., Ashrafinia S., Bakas S., Beukinga R.J., Boellaard R. (2020). The Image Biomarker Standardization Initiative: Standardized Quantitative Radiomics for High-Throughput Image-based Phenotyping. Radiology.

[49] Haralick R.M. (1979). Statistical and Structural Approaches to Texture. Proc. IEEE.

[50] Haralick R.M., Shanmugam K., Dinstein I. (1973). Textural Features for Image Classification. IEEE Trans. Syst. Man Cybern..

[51] Amadasun M., King R. (1989). Textural Features Corresponding to Textural Properties. IEEE Trans. Syst. Man Cybern..

[52] Galloway M.M. (1975). Texture analysis using gray level run lengths. Comput. Graph. Image Process..

[53] Thibault G., Fertil B., Navarro C., Pereira S., Cau P., Levy N., Sequeira J., Mari J.L. (2013). Shape and Texture Indexes Application to Cell Nuclei Classification. Int. J. Pattern Recogn..

[54] Van Griethuysen J.J.M., Fedorov A., Parmar C., Hosny A., Aucoin N., Narayan V., Beets-Tan R.G.H., Fillion-Robin J.C., Pieper S., Aerts H. (2017). Computational Radiomics System to Decode the Radiographic Phenotype. Cancer Res..

[55] Adalsteinsson V.A., Ha G., Freeman S.S., Choudhury A.D., Stover D.G., Parsons H.A., Gydush G., Reed S.C., Rotem D., Rhoades J. (2017). Scalable whole-exome sequencing of cell-free DNA reveals high concordance with metastatic tumors. Nat. Commun..

[56] Wan J.C.M. (2019). Monitoring Trace Levels of ctDNA Using Integration of Variant Reads. Ph.D. Thesis.

[57] R Core Team (2019). R: A Language and Environment for Statistical Computing.

[58] Pinheiro J.C., Bates D.M. (2000). Mixed-Effects Models in S and S-PLUS.

[59] Koller M. (2016). robustlmm: An R Package for Robust Estimation of Linear Mixed-Effects Models. J. Stat. Softw..

[60] Benjamini Y., Hochberg Y. (1995). Controlling the False Discovery Rate—A Practical and Powerful Approach to Multiple Testing. J. R. Stat. Soc. B.

[61] Nakagawa S., Johnson P.C.D., Schielzeth H. (2017). The coefficient of determination R-2 and intra-class correlation coefficient from generalized linear mixed-effects models revisited and expanded. J. R. Soc. Interface.

[62] Schelldorfer J., Buhlmann P., Van De Geer S. (2011). Estimation for High-Dimensional Linear Mixed-Effects Models Using l(1)-Penalization. Scand. J. Stat..

[63] Tibshirani R. (1996). Regression shrinkage and selection via the Lasso. J. Roy. Stat. Soc. B.

[64] Campbell N.A., Lopuhaa H.P., Rousseeuw P.J. (1998). On the calculation of a robust S-estimator of a covariance matrix. Stat. Med..

