# Supplementary material for: Correlating Radiomic Features of Heterogeneity on CT with Circulating Tumor DNA in Metastatic Melanoma
Source: Cancers (Basel). 2020 Nov 24;12(12):3493. doi: 10.3390/cancers12123493 (PMC7759931; doi:10.3390/cancers12123493)
Supplement: Supplementary file 1 [file cancers-12-03493-s001.pdf]

Supplementary Material: Correlating Radiomic Features of Heterogeneity on CT with Circulating Tumor DNA in Metastatic Melanoma

Andrew B Gill, Leonardo Rundo, Jonathan C. M. Wan, Doreen Lau, Jeries P. Zawaideh, Ramona Woitek, Fulvio Zaccagna, Lucian Beer, Davina Gale, Evis Sala, Dominique-Laurent Couturier, Pippa G. Corrie, Nitzan Rosenfeld and Ferdia A. Gallagher

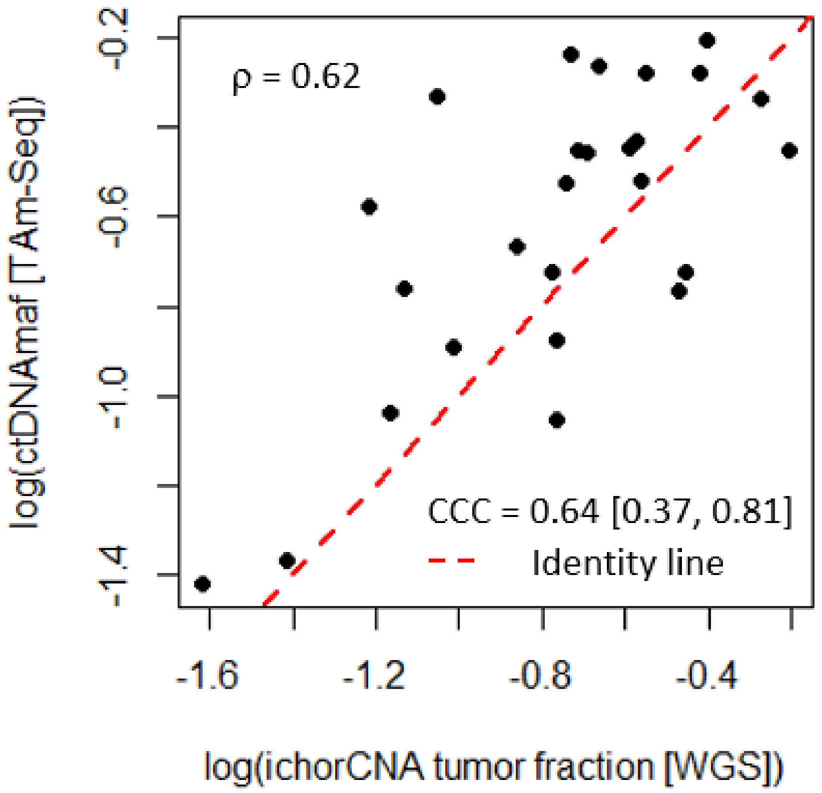

Figure S1. Scatterplot showing concordance between shallow WGS ichorCNA tumor fraction and TAm-Seq ctDNA*maf* measurements, where samples were acquired on the same day. Values less than 0.03 were omitted from this plot since WGS loses sensitivity at this threshold. Statistics shown are concordance correlation coefficient (CCC) and Spearman's rho.

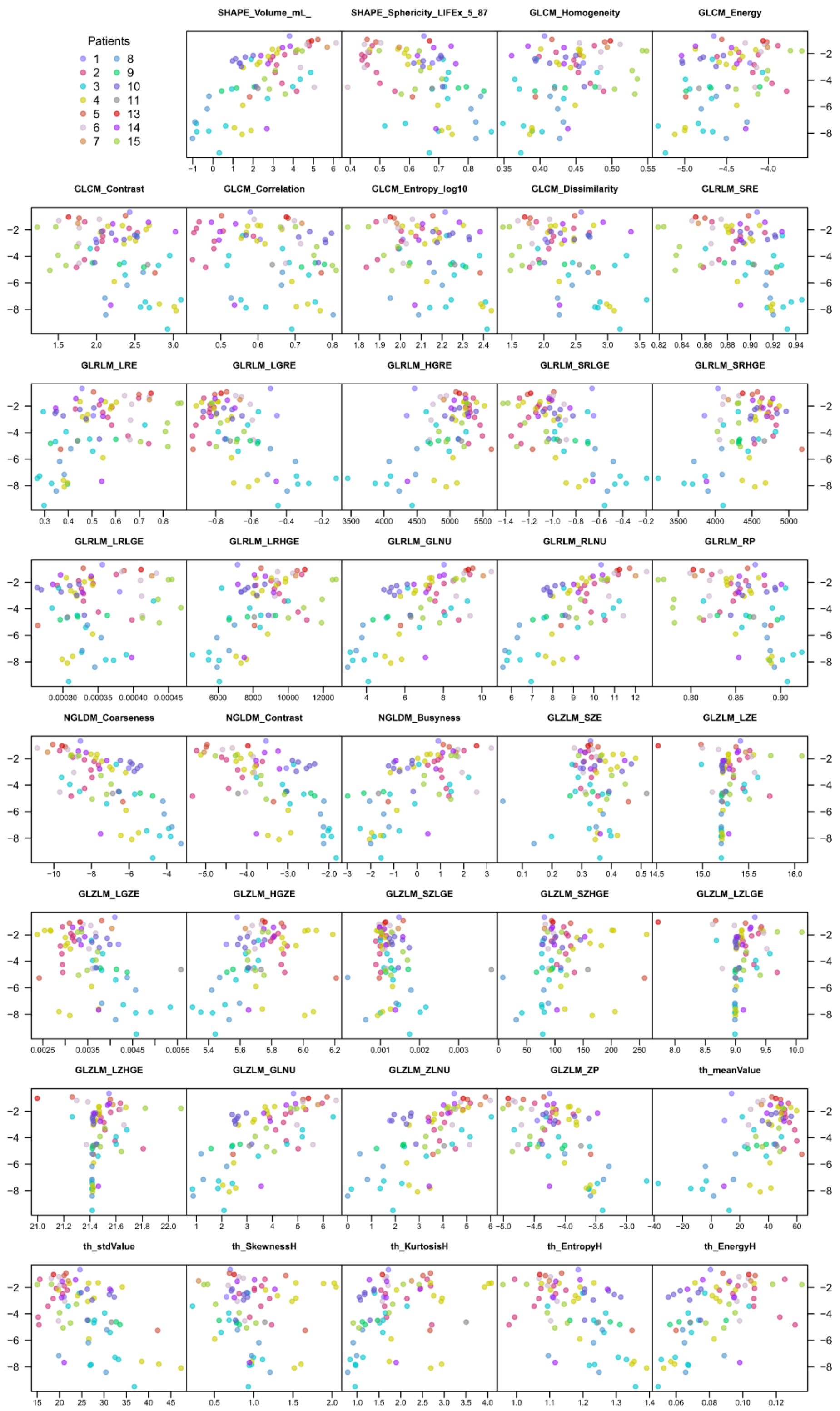

Figure S2. The relationship between each radiomic feature, after the scale transformation, [*x*-axes] and log(ctDNA*maf*) [*y*-axis], color-coded by patient.

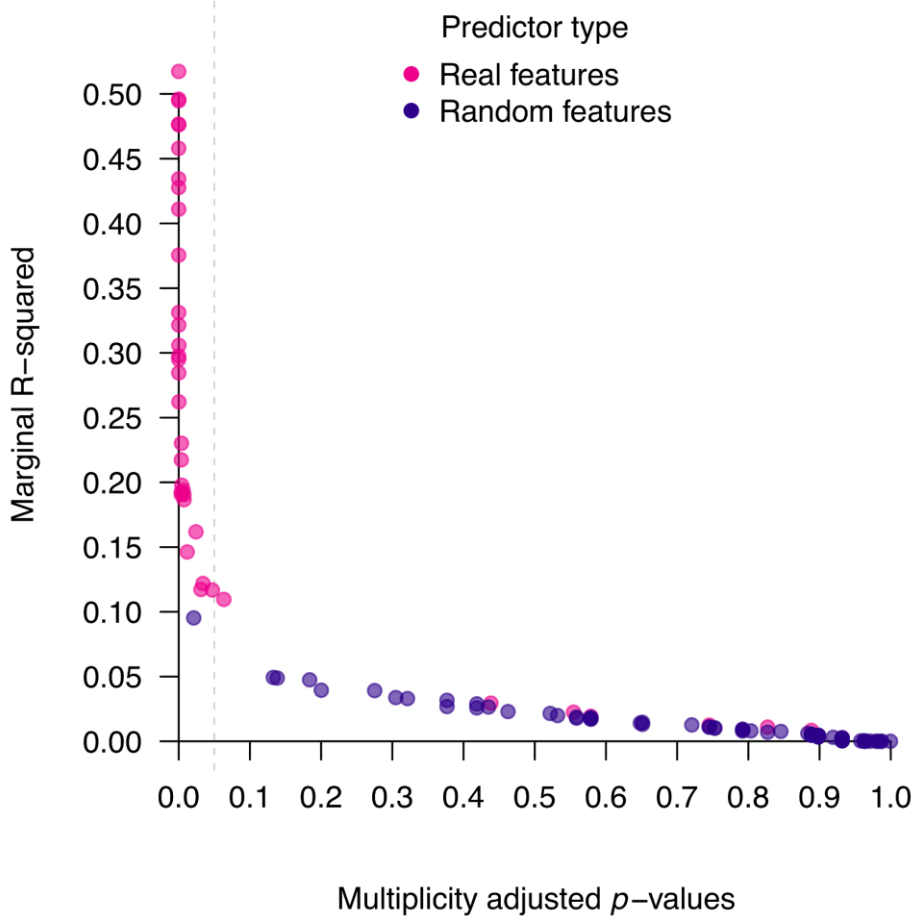

Figure S3. Marginal R-squared (y-axis) plotted with multiplicity adjusted p-values (x-axis), colour-coded by feature type, when explaining log(ctDNA*maf*) without controlling for lesion volume. Using a 5% FDR multiplicity correction, only one random feature over 100 was found to have a significant predictive value: this supports the correctness of the multiplicity correction applied.

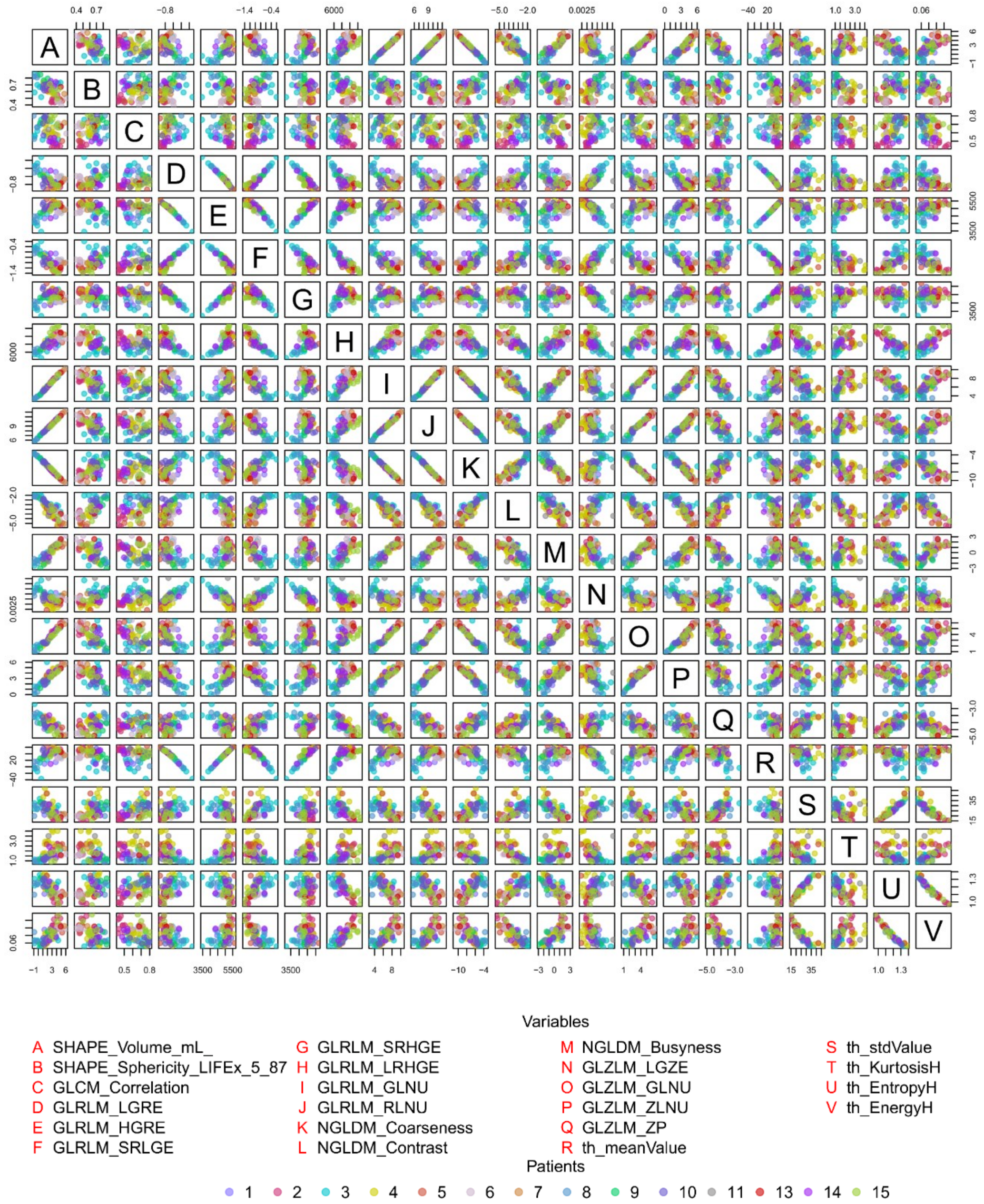

Figure S4. Matrix scatter plot of the radiomic features that showed a significant relationship with ctDNA*maf*, with observations colour coded by patient. This shows a considerable degree of inter-correlation between these features.

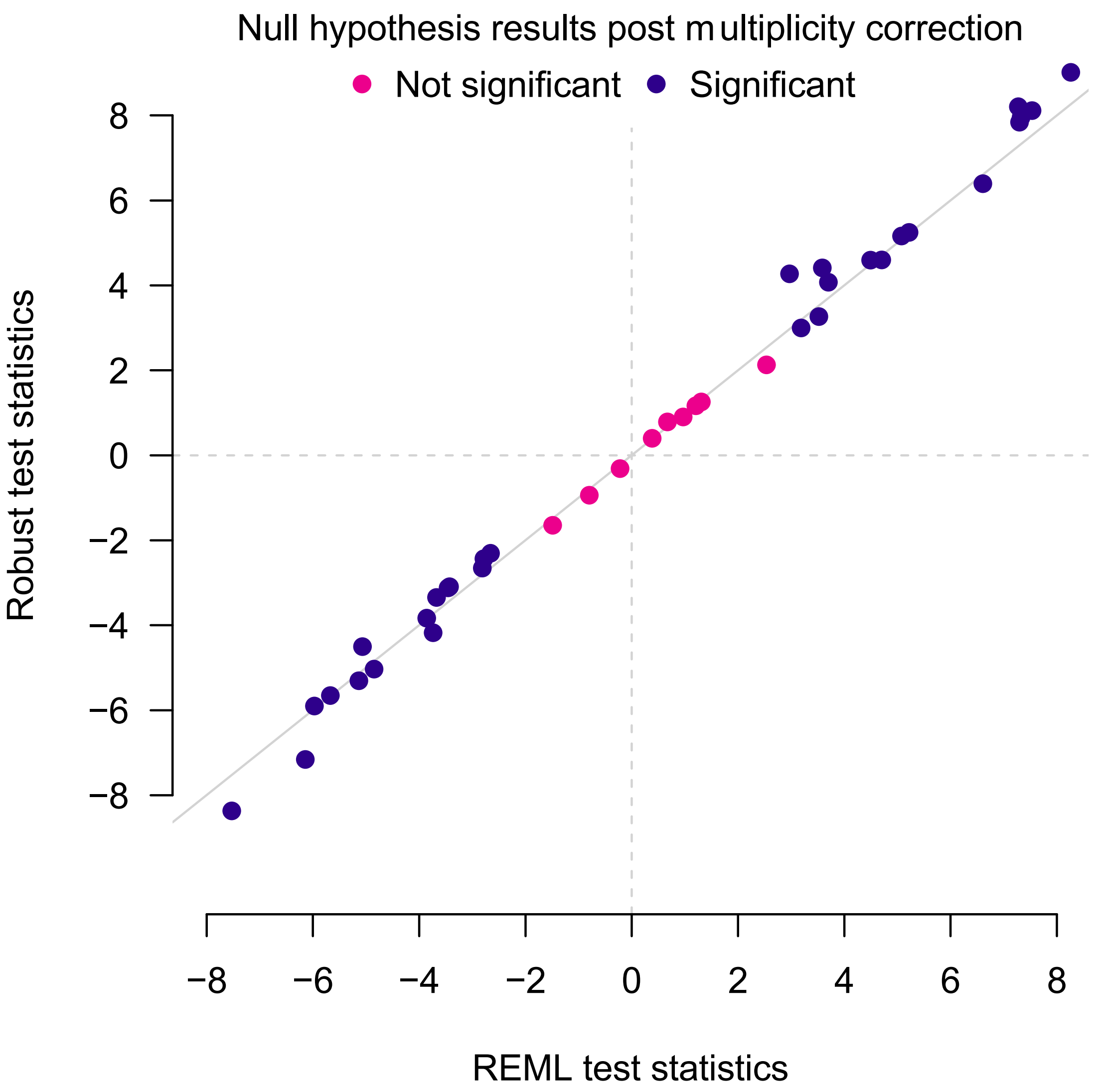

Figure S5. Comparison of the test statistics of the 39 features of interest when fitting feature by feature by means of REML (x-axis) and a robust estimator (y-axis), color coded by multiplicity adjusted p-values.

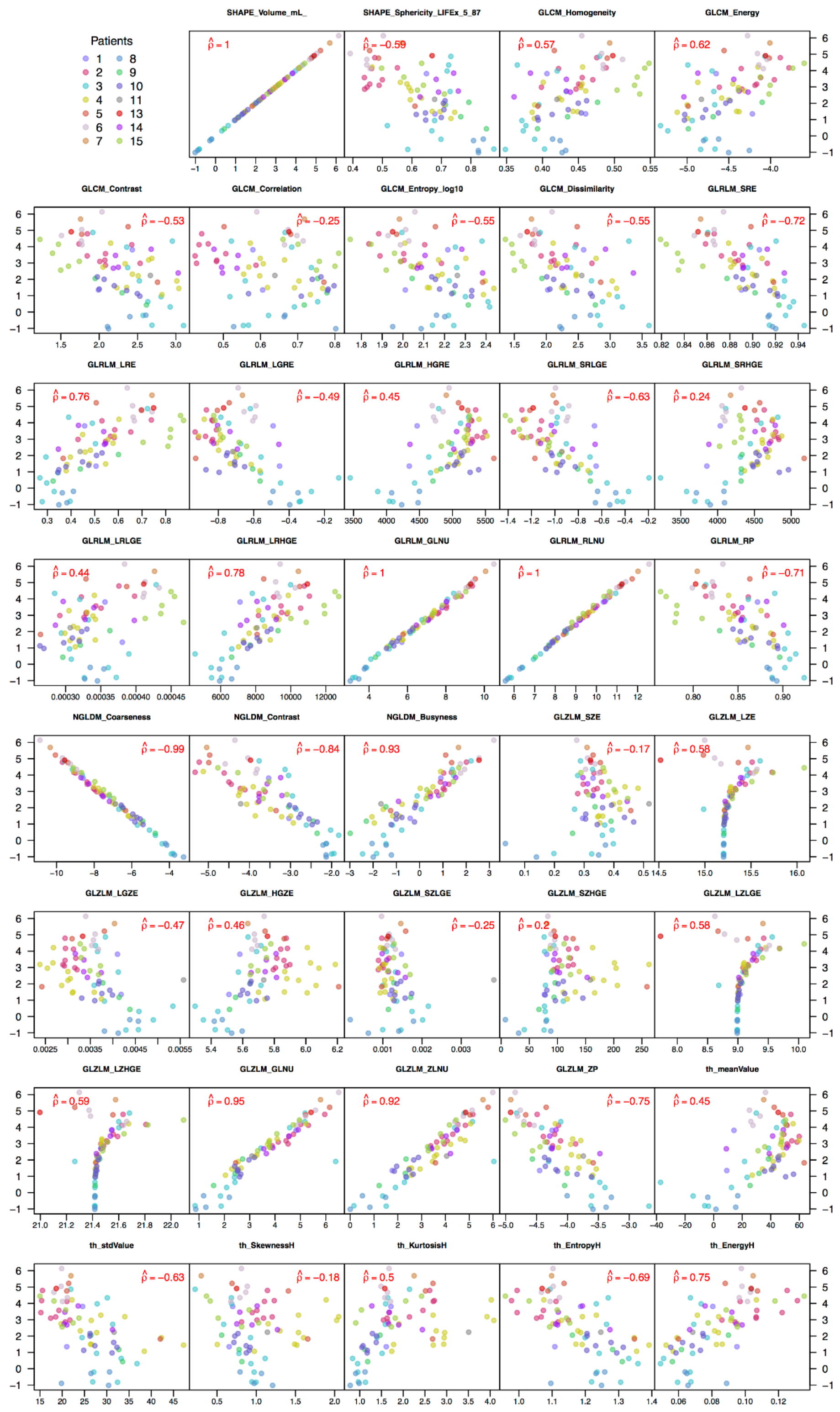

Figure S6. The relationship between each feature (x-axes) and lesion volume (y-axis), color-coded by patient. The Spearman's ρ statistic is shown to indicate the degree of correlation.

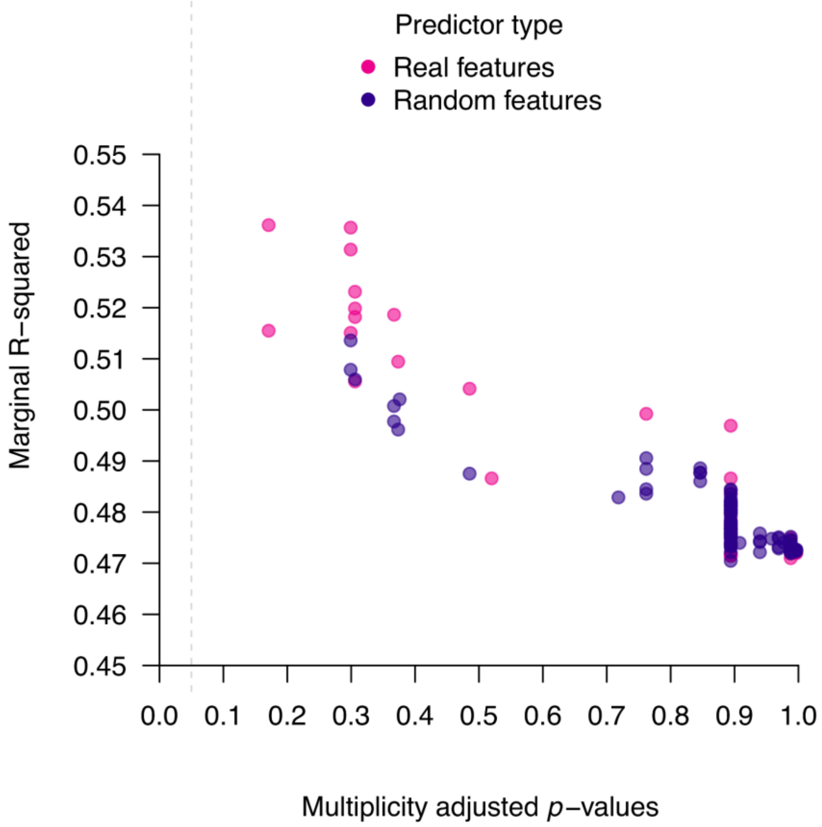

Figure S7. Marginal R-squared (y-axis) plotted against multiplicity adjusted p-values (x-axis), color-coded by feature type, when explaining log(ctDNA*maf*) in addition to the effect of lesion volume. This shows a similar distribution for both real and random features although the two 'best' features are real. These, however, do not yield a significant multiplicity corrected p-value.

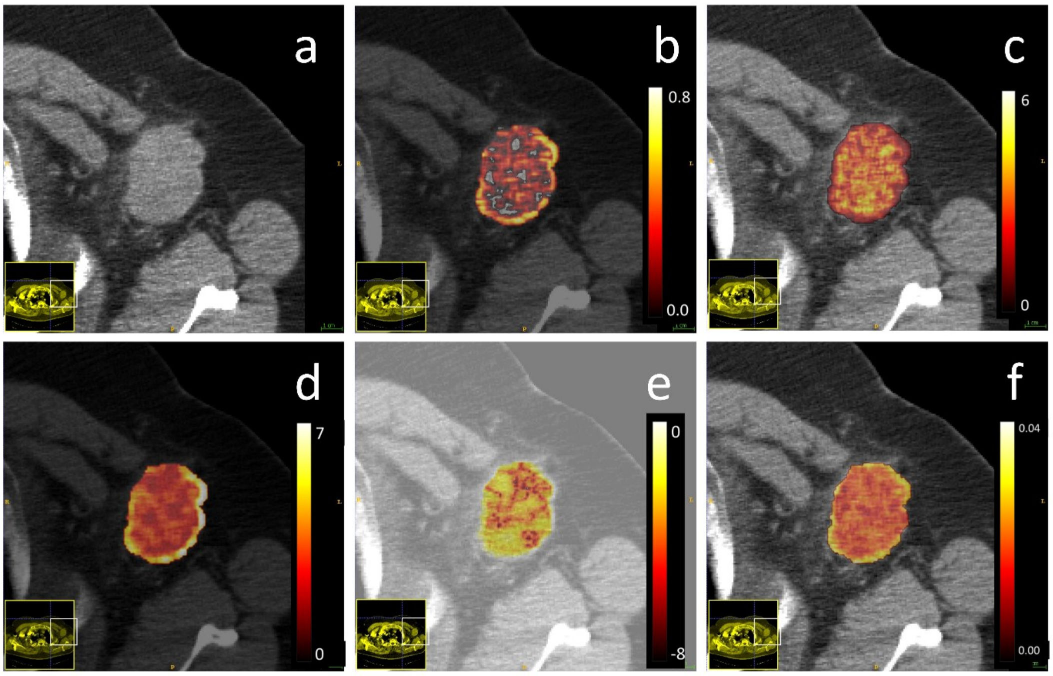

Figure S8. Sample radiomic feature maps within a large tumor (from patient 3). (**a**) Axial CT image; (**b**) map of the 'Correlation' feature; (**c**) the 'GLNUz' feature; (**d**) local 'StdDev'; (**e**) 'log(log(LGRE) + 9)'; and (**f**) the 'Coarseness' feature. All maps were generated with a kernel radius of 1 voxel and features chosen and their order correspond to their importance in the radiomics signature for

ctDNA*maf*. The scale bar in each case shows the dimensionless numerical range of feature values displayed.

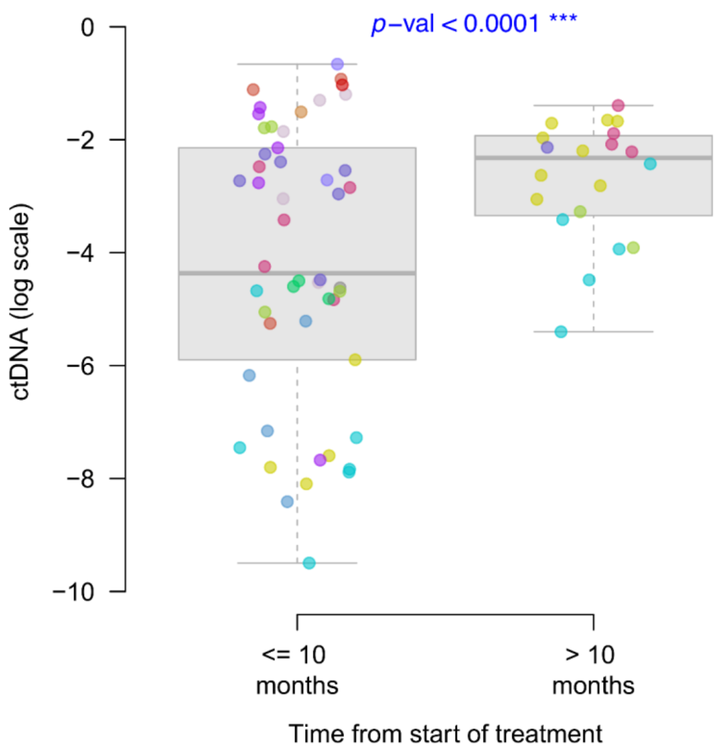

Figure S9. ctDNA with time from treatment start. ctDNAmaf (on the log scale, *y*-axis) as a function of time from start of treatment as a dichotomous variable with observation color coded by patients. Data were modelled by means of a random intercept mixed model. This shows that the average ctDNA*maf* level increases with time while its spread (variance) decreases. *** denotes $p < 0.001$.

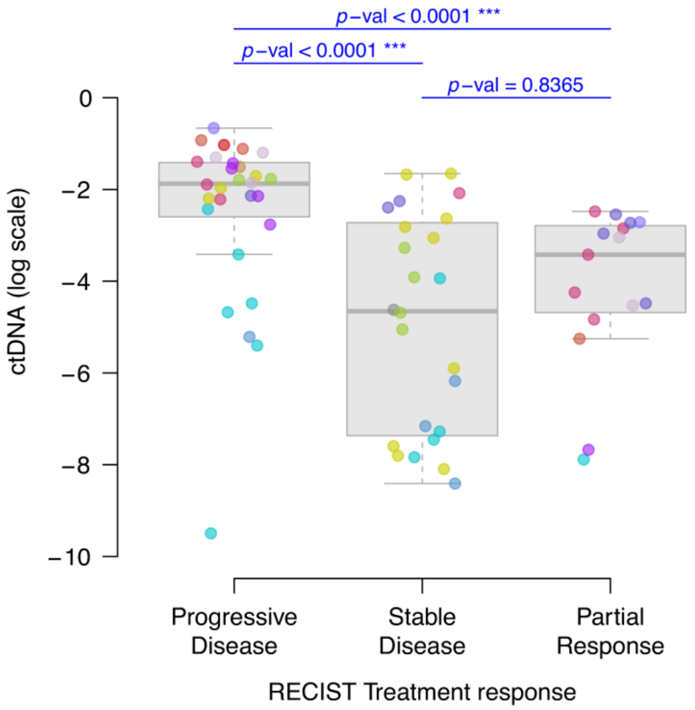

Figure S10. ctDNAmaf on a log scale compared to RECIST treatment response. Higher ctDNA*maf* levels are associated with progressive disease. *** denotes $p < 0.001$.

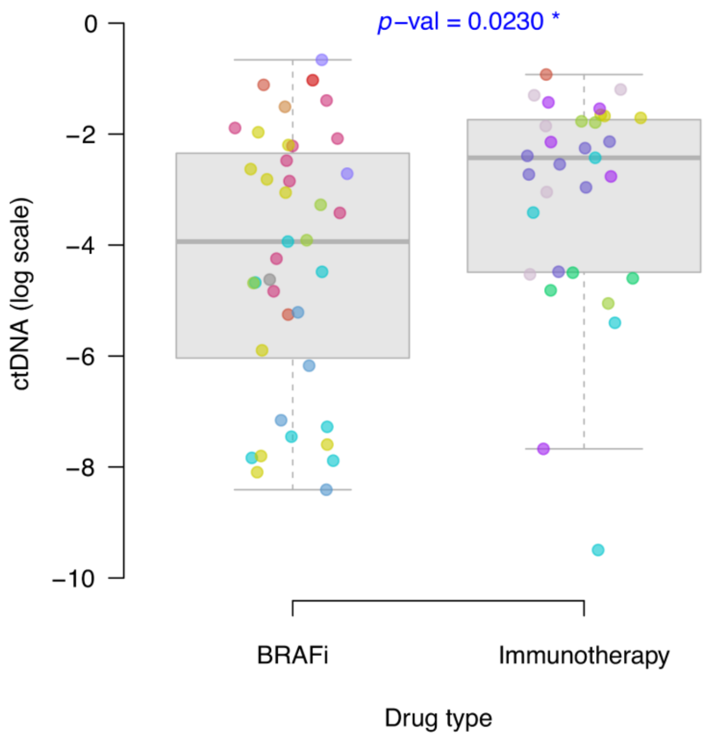

Figure S11. ctDNA*maf* (on the log scale, y-axis) as a function of treatment drug type, where observations are color coded by patient. Data were fitted by means of a random intercept linear mixed model with patients as random effects (which take the with-patient dependence into account) and treatment type as a two-level fixed factor. The *p*-value of the test comparing the mean ctDNA levels per drug type is indicated. Results suggest that the ctDNA levels of patients under immunotherapy were on average higher compared to the ones of patients under BRAFi. Patients with more advanced stages of disease received immunotherapy. * denotes $p < 0.05$.

Table S1. ctDNA assays listed by patient blood sample time-points.

Patient	Blood Sample Time-Point	Mutation	TAm-Seq Mutant Allele Fraction	sWGS Tumour Fraction (ichorCNA)
1	1	BRAF	6.26×10^{-1}	3.92×10^{-1}
	2		7.21×10^{-3}	7.97×10^{-3}
	3		1.60×10^{-1}	
	4		2.16×10^{-1}	1.38×10^{-1}
2	1	BRAF		5.32×10^{-2}
	1			7.87×10^{-2}
	2			1.13×10^{-2}
	3			7.23×10^{-3}
	4			9.68×10^{-3}
	5			1.43×10^{-2}
	20			1.51×10^{-1}
	21			4.19×10^{-2}
	22			2.64×10^{-1}
	22			2.72×10^{-1}
3	1	BRAF	4.87×10^{-5}	9.83×10^{-3}
	2		2.15×10^{-4}	
	3		3.88×10^{-4}	1.12×10^{-2}
	4		7.60×10^{-4}	
	5		5.40×10^{-4}	
	6		5.27×10^{-4}	
	7		7.04×10^{-4}	3.44×10^{-2}
	8		7.19×10^{-4}	
	9		3.70×10^{-4}	
	10		8.10×10^{-4}	
	11		4.79×10^{-5}	1.03×10^{-2}
	12		9.57×10^{-4}	

	13		6.12×10^{-4}	1.87×10^{-2}
	14		2.49×10^{-4}	
	15		7.88×10^{-4}	
	16		6.37×10^{-3}	6.98×10^{-3}
	17		1.87×10^{-2}	2.24×10^{-2}
	18		3.28×10^{-2}	
	19		1.53×10^{-1}	
	20		2.68×10^{-1}	
	21		3.30×10^{-1}	
	22		4.65×10^{-1}	8.88×10^{-2}
4	1	BRAF	4.09×10^{-4}	8.71×10^{-3}
	2		4.43×10^{-4}	
	3		3.74×10^{-3}	
	4		2.09×10^{-4}	
	5		5.97×10^{-4}	3.51×10^{-2}
	6		3.45×10^{-4}	
	7		7.10×10^{-4}	1.59×10^{-2}
	8		2.17×10^{-4}	
	9		8.44×10^{-2}	
	10		4.44×10^{-4}	
	11		3.67×10^{-3}	
	12		2.02×10^{-4}	
	13		9.35×10^{-2}	
	14		8.38×10^{-2}	
	15		2.49×10^{-2}	
	16		6.73×10^{-2}	
	17		6.17×10^{-2}	
	18		7.77×10^{-2}	
	19		9.12×10^{-2}	
	20		1.74×10^{-1}	7.39×10^{-2}
	21		3.62×10^{-1}	
	22		3.54×10^{-1}	1.93×10^{-1}
	23		1.32×10^{-1}	
	24		2.30×10^{-1}	
	25		2.99×10^{-1}	1.81×10^{-1}
5	1	BRAF	3.03×10^{-1}	2.74×10^{-1}
	2		2.39×10^{-3}	9.72×10^{-3}
	3		2.34×10^{-3}	
	4		5.45×10^{-3}	1.95×10^{-2}
	5		1.87×10^{-1}	
	6		3.70×10^{-1}	
	7		3.83×10^{-1}	
	8		5.28×10^{-1}	3.80×10^{-1}
6	1	BRAF	3.59×10^{-1}	2.56×10^{-1}
	2		1.81×10^{-2}	3.81×10^{-2}
	3		8.89×10^{-3}	1.54×10^{-2}
	4		1.17×10^{-2}	
	5		1.06×10^{-1}	
	6		9.20×10^{-2}	6.78×10^{-2}
	7		3.51×10^{-1}	2.03×10^{-1}
	8		5.24×10^{-1}	2.82×10^{-1}
	9		3.72×10^{-1}	2.68×10^{-1}
7	1			3.37×10^{-1}
	2			1.50×10^{-2}
	3			6.78×10^{-2}
	4			2.21×10^{-1}
8	1	BRAF	3.79×10^{-2}	2.42×10^{-2}
	2		1.29×10^{-1}	9.73×10^{-2}
	3		2.41×10^{-4}	8.88×10^{-3}
	4		2.19×10^{-4}	
	5		2.92×10^{-3}	9.41×10^{-3}
	6		4.22×10^{-4}	
	7		5.42×10^{-3}	1.54×10^{-2}
	8		4.12×10^{-4}	2.39×10^{-2}
	9		3.17×10^{-4}	

	10		6.56×10^{-3}	
9	1			9.16×10^{-3}
	2			1.05×10^{-2}
	3			1.18×10^{-2}
10	1			2.22×10^{-2}
	2			3.90×10^{-2}
	3			1.11×10^{-2}
	4			1.14×10^{-2}
	5			4.69×10^{-2}
	16			1.31×10^{-1}
	17			1.22×10^{-1}
11	1			7.14×10^{-2}
	3			1.19×10^{-2}
	5			7.76×10^{-3}
	7			2.22×10^{-2}
12				2.92×10^{-2}
13	1	BRAF	1.89×10^{-1}	3.49×10^{-1}
	2		8.11×10^{-2}	
	3		1.72×10^{-1}	3.36×10^{-1}
	4		3.54×10^{-1}	6.22×10^{-1}
	5		2.48×10^{-1}	
	6		4.61×10^{-1}	5.30×10^{-1}
14	1	BRAF	1.33×10^{-1}	1.71×10^{-1}
	2		2.42×10^{-4}	9.02×10^{-3}
	3		5.02×10^{-4}	9.87×10^{-3}
	4		3.03×10^{-3}	7.24×10^{-2}
	5		2.65×10^{-1}	6.05×10^{-2}
	6		5.80×10^{-1}	1.85×10^{-1}
	7		5.47×10^{-1}	2.16×10^{-1}
	7			2.26×10^{-1}
15	1	NRAS	8.84×10^{-2}	1.71×10^{-1}
	2		1.89×10^{-1}	1.67×10^{-1}
	3		2.32×10^{-2}	4.33×10^{-2}
	4		3.85×10^{-2}	
	5		4.96×10^{-3}	
	6		6.69×10^{-3}	
	7		7.67×10^{-4}	
	8		1.47×10^{-2}	
	9		2.74×10^{-4}	3.21×10^{-2}
	10		1.69×10^{-3}	
	11		8.90×10^{-3}	9.69×10^{-3}
	12		5.43×10^{-2}	
	13		4.30×10^{-2}	3.85×10^{-2}

Assays were by TAm-Seq mutant allele fraction (targeting the mutation shown) and/or sWGS carried out by ichorCNA.

Table S2. Interpolated ctDNA*maf* readings listed by patient CT imaging visit.

Patient	CT Imaging Time-Point	Assay	Interpolated ctDNAmaf
1	i	ichorCNA	5.16×10^{-1}
	ii	ichorCNA	6.63×10^{-2}
2	i	ichorCNA	7.96×10^{-3}
	ii	ichorCNA	1.43×10^{-2}
	iii	ichorCNA	3.27×10^{-2}
	iv	ichorCNA	5.80×10^{-2}
	v	ichorCNA	8.39×10^{-2}
	vi	ichorCNA	1.09×10^{-1}
	vii	ichorCNA	1.25×10^{-1}
	viii	ichorCNA	1.51×10^{-1}
	ix	ichorCNA	2.48×10^{-1}
3	i	ichorCNA	9.32×10^{-3}
	ii	TAm-Seq	3.76×10^{-4}
	iii	TAm-Seq	5.79×10^{-4}
	iv	TAm-Seq	6.92×10^{-4}
	v	TAm-Seq	3.95×10^{-4}

	vi	TAm−Seq	7.51×10^{-5}
	vii	TAm−Seq	4.51×10^{-3}
	viii	TAm−Seq	1.13×10^{-2}
	ix	ichorCNA	1.95×10^{-2}
	x	ichorCNA	3.29×10^{-2}
	xi	ichorCNA	8.83×10^{-2}
4	i	TAm−Seq	4.09×10^{-4}
	ii	TAm−Seq	5.02×10^{-4}
	iii	TAm−Seq	3.05×10^{-4}
	iv	TAm−Seq	2.75×10^{-3}
	v	ichorCNA	4.71×10^{-2}
	vi	ichorCNA	5.99×10^{-2}
	vii	ichorCNA	7.20×10^{-2}
	viii	ichorCNA	1.11×10^{-1}
	ix	ichorCNA	1.40×10^{-1}
	x	ichorCNA	1.88×10^{-1}
	xi	ichorCNA	1.91×10^{-1}
	xii	ichorCNA	1.81×10^{-1}
5	i	TAm−Seq	5.23×10^{-3}
	ii	ichorCNA	3.29×10^{-1}
	iii	ichorCNA	3.96×10^{-1}
6	i	ichorCNA	3.02×10^{-1}
	ii	TAm−Seq	1.08×10^{-2}
	iii	ichorCNA	4.76×10^{-2}
	iv	ichorCNA	1.57×10^{-1}
	v	ichorCNA	2.72×10^{-1}
7	i	ichorCNA	2.21×10^{-1}
8	i	n/a	n/a
	ii	TAm−Seq	2.23×10^{-4}
	iii	TAm−Seq	7.79×10^{-4}
	iv	TAm−Seq	2.08×10^{-3}
	v	TAm−Seq	5.45×10^{-3}
9	1	ichorCNA	8.10×10^{-3}
	2	ichorCNA	1.01×10^{-2}
	3	ichorCNA	1.11×10^{-2}
10	i	ichorCNA	1.13×10^{-2}
	ii	ichorCNA	5.18×10^{-2}
	iii	ichorCNA	6.54×10^{-2}
	iv	ichorCNA	7.85×10^{-2}
	v	ichorCNA	9.14×10^{-2}
	vi	ichorCNA	1.05×10^{-1}
	vii	ichorCNA	1.18×10^{-1}
11	i	ichorCNA	9.82×10^{-3}
12	i	n/a	n/a
13	i	ichorCNA	3.37×10^{-1}
14	i	ichorCNA	1.17×10^{-1}
	iii	TAm−Seq	4.65×10^{-4}
	iii	ichorCNA	6.31×10^{-2}
	iv	ichorCNA	2.14×10^{-1}
	v	ichorCNA	2.40×10^{-1}
15	i	ichorCNA	1.70×10^{-1}
	ii	ichorCNA	1.67×10^{-1}
	iii	TAm−Seq	6.39×10^{-3}
	iv	TAm−Seq	9.24×10^{-3}
	v	ichorCNA	2.00×10^{-2}
	vi	ichorCNA	3.78×10^{-2}

Interpolation was linear by time interval between values listed in Table S1a. The sWGS ichorCNA assay was used except when a TAm-Seq value was available and was less than the sWGS sensitivity threshold (<0.03).

Table S3. Statistics from feature-by-feature mixed linear models predicting ctDNA*maf* without controlling for lesion volume.

Feature	*t*-Value	*p*-Value	adj. *p*-Value	Sig.	R-Squared
GLNUz	8.263	1.11×10^{-11}	1.54×10^{-9}	***	0.517
GLNUr	7.531	7.49×10^{-10}	2.80×10^{-8}	***	0.496
Coarseness	−7.527	6.86×10^{-10}	2.80×10^{-8}	***	0.495
ZLNU	7.273	8.06×10^{-10}	2.80×10^{-8}	***	0.458
RLNU	7.327	1.43×10^{-9}	3.98×10^{-8}	***	0.477
Volume	7.295	1.79×10^{-9}	4.16×10^{-8}	***	0.476
Busyness	6.608	1.57×10^{-8}	3.12×10^{-7}	***	0.428
Contrast	−6.143	2.81×10^{-7}	4.89×10^{-6}	***	0.434
SRLGE	−5.973	3.75×10^{-7}	5.80×10^{-6}	***	0.411
LGRE	−5.673	7.99×10^{-7}	1.11×10^{-5}	***	0.376
StdDev	−5.137	2.59×10^{-6}	3.27×10^{-5}	***	0.295
Mean	5.218	4.24×10^{-6}	4.92×10^{-5}	***	0.331
ZP	−5.069	4.67×10^{-6}	5.00×10^{-5}	***	0.298
HGRE	5.075	6.81×10^{-6}	6.76×10^{-5}	***	0.321
Entropy_h	−4.85	8.19×10^{-6}	7.59×10^{-5}	***	0.285
LRHGE	4.703	3.04×10^{-5}	2.65×10^{-4}	***	0.306
Energy	4.494	3.30×10^{-5}	2.70×10^{-4}	***	0.262
Correlation	−3.676	4.80×10^{-4}	3.51×10^{-3}	**	0.191
SRHGE	3.699	4.75×10^{-4}	3.51×10^{-3}	**	0.192
LGZE	−3.74	5.23×10^{-4}	3.64×10^{-3}	**	0.217
Sphericity	−3.859	5.84×10^{-4}	3.87×10^{-3}	**	0.23
Kurtosis	3.586	6.84×10^{-4}	4.32×10^{-3}	**	0.198
LRE	3.522	1.04×10^{-3}	6.26×10^{-3}	**	0.194
SRE	−3.456	1.26×10^{-3}	7.29×10^{-3}	**	0.191
RP	−3.428	1.35×10^{-3}	7.53×10^{-3}	**	0.187
Uniformity	3.189	2.23×10^{-3}	1.19×10^{-2}	*	0.146
Random35	2.975	4.09×10^{-3}	2.11×10^{-2}	*	0.095
HGZE	2.969	4.88×10^{-3}	2.42×10^{-2}	*	0.162
Entropy	−2.813	6.51×10^{-3}	3.12×10^{-2}	*	0.117
Dissimilarity	−2.784	7.35×10^{-3}	3.41×10^{-2}	*	0.122
Contrast	−2.657	1.05×10^{-2}	4.72×10^{-2}	*	0.117
Homogeneity	2.534	1.46×10^{-2}	6.34×10^{-2}		0.11
Random36	2.202	3.15×10^{-2}	1.33×10^{-1}		0.049
Random77	2.171	3.38×10^{-2}	1.38×10^{-1}		0.049
Random80	−2.032	4.63×10^{-2}	1.84×10^{-1}		0.047
Random78	−1.984	5.19×10^{-2}	2.00×10^{-1}		0.039
Random79	−1.82	7.33×10^{-2}	2.75×10^{-1}		0.039
Random91	−1.759	8.34×10^{-2}	3.05×10^{-1}		0.034
Random81	1.721	9.01×10^{-2}	3.21×10^{-1}		0.033
Random19	1.615	1.11×10^{-1}	3.77×10^{-1}		0.032
Random45	1.617	1.11×10^{-1}	3.77×10^{-1}		0.027
Random17	1.535	1.30×10^{-1}	4.19×10^{-1}		0.029
Random30	1.538	1.29×10^{-1}	4.19×10^{-1}		0.026
Random50	−1.503	1.38×10^{-1}	4.35×10^{-1}		0.026
SZLGE	−1.487	1.42×10^{-1}	4.39×10^{-1}		0.029
Random83	1.446	1.53×10^{-1}	4.63×10^{-1}		0.023
Random7	1.367	1.76×10^{-1}	5.22×10^{-1}		0.022
Random69	−1.345	1.84×10^{-1}	5.32×10^{-1}		0.02
LZHGE	1.307	1.96×10^{-1}	5.55×10^{-1}		0.022
Random61	1.283	2.04×10^{-1}	5.59×10^{-1}		0.018
Random63	1.281	2.05×10^{-1}	5.59×10^{-1}		0.019
LZE	1.209	2.31×10^{-1}	5.79×10^{-1}		0.019
Random27	1.24	2.20×10^{-1}	5.79×10^{-1}		0.018
Random59	−1.236	2.21×10^{-1}	5.79×10^{-1}		0.018
Random85	−1.203	2.33×10^{-1}	5.79×10^{-1}		0.018
Random99	−1.222	2.26×10^{-1}	5.79×10^{-1}		0.017
Random100	1.123	2.66×10^{-1}	6.48×10^{-1}		0.014
Random84	1.102	2.75×10^{-1}	6.51×10^{-1}		0.013
Random87	−1.097	2.77×10^{-1}	6.51×10^{-1}		0.015
Random76	1.021	3.11×10^{-1}	7.21×10^{-1}		0.013
LZLGE	0.966	3.38×10^{-1}	7.45×10^{-1}		0.012

Random24	0.968	3.37×10^{-1}	7.45×10^{-1}	0.011
Random28	0.972	3.35×10^{-1}	7.45×10^{-1}	0.011
Random46	0.941	3.50×10^{-1}	7.53×10^{-1}	0.01
Random62	0.938	3.52×10^{-1}	7.53×10^{-1}	0.01
Random14	0.872	3.87×10^{-1}	7.92×10^{-1}	0.009
Random29	−0.867	3.89×10^{-1}	7.92×10^{-1}	0.008
Random32	−0.88	3.82×10^{-1}	7.92×10^{-1}	0.009
Random88	−0.86	3.93×10^{-1}	7.92×10^{-1}	0.009
Random48	0.839	4.05×10^{-1}	8.04×10^{-1}	0.008
Skewness	−0.8	4.27×10^{-1}	8.27×10^{-1}	0.011
Random68	−0.797	4.28×10^{-1}	8.27×10^{-1}	0.007
Random64	0.77	4.44×10^{-1}	8.46×10^{-1}	0.008
Random15	−0.726	4.70×10^{-1}	8.84×10^{-1}	0.006
SZHGE	0.67	5.06×10^{-1}	8.89×10^{-1}	0.008
Random31	0.677	5.01×10^{-1}	8.89×10^{-1}	0.005
Random39	0.657	5.14×10^{-1}	8.89×10^{-1}	0.005
Random54	0.65	5.18×10^{-1}	8.89×10^{-1}	0.005
Random60	0.658	5.13×10^{-1}	8.89×10^{-1}	0.005
Random73	−0.686	4.95×10^{-1}	8.89×10^{-1}	0.005
Random96	0.696	4.89×10^{-1}	8.89×10^{-1}	0.005
Random1	−0.535	5.95×10^{-1}	8.98×10^{-1}	0.004
Random3	−0.544	5.89×10^{-1}	8.98×10^{-1}	0.003
Random4	−0.597	5.53×10^{-1}	8.98×10^{-1}	0.004
Random5	−0.606	5.47×10^{-1}	8.98×10^{-1}	0.005
Random16	−0.613	5.42×10^{-1}	8.98×10^{-1}	0.005
Random22	−0.546	5.87×10^{-1}	8.98×10^{-1}	0.003
Random47	−0.596	5.54×10^{-1}	8.98×10^{-1}	0.004
Random55	−0.576	5.67×10^{-1}	8.98×10^{-1}	0.004
Random90	−0.582	5.63×10^{-1}	8.98×10^{-1}	0.004
Random94	0.562	5.76×10^{-1}	8.98×10^{-1}	0.004
Random98	0.567	5.73×10^{-1}	8.98×10^{-1}	0.004
Random6	0.505	6.15×10^{-1}	9.19×10^{-1}	0.003
LRLGE	0.382	7.04×10^{-1}	9.32×10^{-1}	0.003
SZE	−0.221	8.26×10^{-1}	9.32×10^{-1}	0.001
Random2	−0.265	7.92×10^{-1}	9.32×10^{-1}	0.001
Random8	0.324	7.47×10^{-1}	9.32×10^{-1}	0.001
Random10	0.437	6.64×10^{-1}	9.32×10^{-1}	0.003
Random11	−0.434	6.66×10^{-1}	9.32×10^{-1}	0.002
Random12	0.4	6.90×10^{-1}	9.32×10^{-1}	0.002
Random13	0.197	8.45×10^{-1}	9.32×10^{-1}	0
Random18	0.39	6.98×10^{-1}	9.32×10^{-1}	0.002
Random20	−0.314	7.54×10^{-1}	9.32×10^{-1}	0.001
Random21	0.319	7.50×10^{-1}	9.32×10^{-1}	0.001
Random25	0.27	7.88×10^{-1}	9.32×10^{-1}	0.001
Random26	−0.213	8.32×10^{-1}	9.32×10^{-1}	0.001
Random33	0.371	7.12×10^{-1}	9.32×10^{-1}	0.002
Random34	0.48	6.33×10^{-1}	9.32×10^{-1}	0.003
Random38	−0.197	8.44×10^{-1}	9.32×10^{-1}	0
Random40	0.373	7.10×10^{-1}	9.32×10^{-1}	0.002
Random41	−0.301	7.64×10^{-1}	9.32×10^{-1}	0.001
Random42	0.366	7.16×10^{-1}	9.32×10^{-1}	0.002
Random43	−0.421	6.75×10^{-1}	9.32×10^{-1}	0.002
Random44	0.4	6.91×10^{-1}	9.32×10^{-1}	0.002
Random49	0.263	7.93×10^{-1}	9.32×10^{-1}	0.001
Random52	−0.255	7.99×10^{-1}	9.32×10^{-1}	0.001
Random53	−0.295	7.69×10^{-1}	9.32×10^{-1}	0.001
Random57	0.304	7.62×10^{-1}	9.32×10^{-1}	0.001
Random70	0.204	8.39×10^{-1}	9.32×10^{-1}	0.001
Random71	0.286	7.76×10^{-1}	9.32×10^{-1}	0.001
Random72	−0.321	7.49×10^{-1}	9.32×10^{-1}	0.001
Random74	0.449	6.55×10^{-1}	9.32×10^{-1}	0.002
Random75	−0.242	8.09×10^{-1}	9.32×10^{-1}	0.001
Random92	−0.349	7.29×10^{-1}	9.32×10^{-1}	0.001
Random93	0.325	7.46×10^{-1}	9.32×10^{-1}	0.001
Random95	−0.201	8.42×10^{-1}	9.32×10^{-1}	0

Random66	0.158	8.75×10^{-1}	9.58×10^{-1}		0
Random23	−0.121	9.04×10^{-1}	9.63×10^{-1}		0
Random37	−0.131	8.97×10^{-1}	9.63×10^{-1}		0
Random65	0.112	9.11×10^{-1}	9.63×10^{-1}		0
Random67	−0.135	8.93×10^{-1}	9.63×10^{-1}		0
Random97	−0.108	9.14×10^{-1}	9.63×10^{-1}		0
Random86	−0.096	9.24×10^{-1}	9.65×10^{-1}		0
Random56	0.08	9.36×10^{-1}	9.71×10^{-1}		0
Random58	0.061	9.52×10^{-1}	9.80×10^{-1}		0
Random89	0.05	9.61×10^{-1}	9.82×10^{-1}		0
Random9	−0.03	9.76×10^{-1}	9.87×10^{-1}		0
Random51	−0.025	9.80×10^{-1}	9.87×10^{-1}		0
Random82	0.001	1.00×10^{0}	1.00×10^{0}		0

*** $p < 0.001$, ** $p < 0.01$, * $p < 0.05$.

Table S4. Statistics from feature-by-feature mixed linear models predicting ctDNA*maf* with lesion volume included as a fixed effect.

Feature	*t*-Value	*p*-Value	adj. *p*-Value	Sig.	R-Squared
Correlation	−3.227	2.03×10^{-3}	1.71×10^{-1}		0.536
GLNUz	3.151	2.48×10^{-3}	1.71×10^{-1}		0.515
StdDev	−2.8	6.68×10^{-3}	2.99×10^{-1}		0.531
LGRE	−2.7	1.05×10^{-2}	2.99×10^{-1}		0.536
Random78	−2.506	1.49×10^{-2}	2.99×10^{-1}		0.514
Coarseness	−2.512	1.50×10^{-2}	2.99×10^{-1}		0.515
Random45	2.497	1.52×10^{-2}	2.99×10^{-1}		0.508
Mean	2.419	2.06×10^{-2}	3.06×10^{-1}		0.523
SRHGE	2.359	2.30×10^{-2}	3.06×10^{-1}		0.518
Random83	2.322	2.34×10^{-2}	3.06×10^{-1}		0.506
LRLGE	−2.3	2.59×10^{-2}	3.06×10^{-1}		0.506
HGRE	2.308	2.66×10^{-2}	3.06×10^{-1}		0.52
Random77	2.135	3.67×10^{-2}	3.67×10^{-1}		0.501
Random80	−2.126	3.72×10^{-2}	3.67×10^{-1}		0.498
SRLGE	−2.131	3.99×10^{-2}	3.67×10^{-1}		0.519
GLNUr	2.034	4.59×10^{-2}	3.74×10^{-1}		0.509
Random91	−2.034	4.60×10^{-2}	3.74×10^{-1}		0.496
Random79	−2.005	4.90×10^{-2}	3.76×10^{-1}		0.502
Random35	1.851	6.87×10^{-2}	4.85×10^{-1}		0.488
Entropy_h	−1.839	7.03×10^{-2}	4.85×10^{-1}		0.504
ZLNU	1.783	7.91×10^{-2}	5.20×10^{-1}		0.487
Random66	1.6	1.15×10^{-1}	7.18×10^{-1}		0.483
LGZE	−1.543	1.30×10^{-1}	7.62×10^{-1}		0.499
Random36	1.519	1.34×10^{-1}	7.62×10^{-1}		0.484
Random61	1.487	1.42×10^{-1}	7.62×10^{-1}		0.484
Random68	−1.467	1.48×10^{-1}	7.62×10^{-1}		0.491
Random62	1.461	1.49×10^{-1}	7.62×10^{-1}		0.488
Random90	−1.359	1.79×10^{-1}	8.46×10^{-1}		0.486
Random72	1.332	1.88×10^{-1}	8.46×10^{-1}		0.488
Random64	1.324	1.90×10^{-1}	8.46×10^{-1}		0.489
Random73	−1.325	1.90×10^{-1}	8.46×10^{-1}		0.488
Random81	1.249	2.16×10^{-1}	8.94×10^{-1}		0.483
Random17	1.168	2.47×10^{-1}	8.94×10^{-1}		0.472
Random69	−1.132	2.62×10^{-1}	8.94×10^{-1}		0.482
Sphericity	−1.126	2.65×10^{-1}	8.94×10^{-1}		0.497
Random47	−1.113	2.70×10^{-1}	8.94×10^{-1}		0.484
Random92	1.098	2.76×10^{-1}	8.94×10^{-1}		0.482
Random20	1.055	2.95×10^{-1}	8.94×10^{-1}		0.484
Random57	1.05	2.98×10^{-1}	8.94×10^{-1}		0.48
Random100	1.047	2.99×10^{-1}	8.94×10^{-1}		0.481
Random15	−1.033	3.05×10^{-1}	8.94×10^{-1}		0.482
Kurtosis	1.031	3.08×10^{-1}	8.94×10^{-1}		0.48
Random99	−1.021	3.11×10^{-1}	8.94×10^{-1}		0.481
Random27	1.021	3.11×10^{-1}	8.94×10^{-1}		0.48
Random95	−1.013	3.15×10^{-1}	8.94×10^{-1}		0.481
Random51	−0.997	3.22×10^{-1}	8.94×10^{-1}		0.48

LRE	−0.998	3.23×10^{-1}	8.94×10^{-1}	0.474
Random14	0.976	3.33×10^{-1}	8.94×10^{-1}	0.484
Random85	−0.974	3.33×10^{-1}	8.94×10^{-1}	0.482
Random49	−0.968	3.37×10^{-1}	8.94×10^{-1}	0.478
Random55	−0.96	3.41×10^{-1}	8.94×10^{-1}	0.478
Random46	0.957	3.42×10^{-1}	8.94×10^{-1}	0.476
Random26	−0.93	3.56×10^{-1}	8.94×10^{-1}	0.477
Random44	−0.922	3.60×10^{-1}	8.94×10^{-1}	0.48
Energy	0.921	3.60×10^{-1}	8.94×10^{-1}	0.484
Contrast	−0.905	3.70×10^{-1}	8.94×10^{-1}	0.487
Random33	−0.9	3.71×10^{-1}	8.94×10^{-1}	0.481
Random24	0.889	3.77×10^{-1}	8.94×10^{-1}	0.478
Random19	0.874	3.86×10^{-1}	8.94×10^{-1}	0.478
SZLGE	−0.83	4.10×10^{-1}	8.94×10^{-1}	0.477
Random93	−0.827	4.12×10^{-1}	8.94×10^{-1}	0.476
HGZE	0.773	4.44×10^{-1}	8.94×10^{-1}	0.481
Random9	−0.765	4.47×10^{-1}	8.94×10^{-1}	0.476
Random5	−0.757	4.52×10^{-1}	8.94×10^{-1}	0.476
LZHGE	−0.743	4.60×10^{-1}	8.94×10^{-1}	0.477
LZE	−0.717	4.76×10^{-1}	8.94×10^{-1}	0.477
Random50	−0.715	4.77×10^{-1}	8.94×10^{-1}	0.473
Random87	−0.715	4.77×10^{-1}	8.94×10^{-1}	0.474
RP	0.71	4.81×10^{-1}	8.94×10^{-1}	0.472
Busyness	0.701	4.87×10^{-1}	8.94×10^{-1}	0.475
Random10	0.692	4.91×10^{-1}	8.94×10^{-1}	0.479
Random22	−0.686	4.95×10^{-1}	8.94×10^{-1}	0.475
Entropy	−0.653	5.16×10^{-1}	8.94×10^{-1}	0.479
LZLGE	−0.648	5.19×10^{-1}	8.94×10^{-1}	0.476
Random63	0.644	5.22×10^{-1}	8.94×10^{-1}	0.47
Random30	0.643	5.22×10^{-1}	8.94×10^{-1}	0.474
Random32	−0.642	5.23×10^{-1}	8.94×10^{-1}	0.475
SRE	0.643	5.23×10^{-1}	8.94×10^{-1}	0.471
Random25	−0.638	5.26×10^{-1}	8.94×10^{-1}	0.473
Random89	−0.624	5.35×10^{-1}	8.94×10^{-1}	0.476
Random13	0.615	5.41×10^{-1}	8.94×10^{-1}	0.477
Random2	0.609	5.45×10^{-1}	8.94×10^{-1}	0.477
Random37	0.604	5.48×10^{-1}	8.94×10^{-1}	0.474
RLNU	0.604	5.49×10^{-1}	8.94×10^{-1}	0.471
Random43	−0.6	5.51×10^{-1}	8.94×10^{-1}	0.477
Random96	0.577	5.66×10^{-1}	9.08×10^{-1}	0.474
Random12	−0.534	5.95×10^{-1}	9.40×10^{-1}	0.474
Random8	0.526	6.01×10^{-1}	9.40×10^{-1}	0.474
Random84	0.512	6.10×10^{-1}	9.40×10^{-1}	0.472
Random88	−0.508	6.13×10^{-1}	9.40×10^{-1}	0.476
Random58	0.481	6.32×10^{-1}	9.58×10^{-1}	0.475
Random48	0.45	6.54×10^{-1}	9.69×10^{-1}	0.473
Random74	0.449	6.55×10^{-1}	9.69×10^{-1}	0.473
Random40	−0.44	6.62×10^{-1}	9.69×10^{-1}	0.475
Random38	0.432	6.67×10^{-1}	9.69×10^{-1}	0.475
Random7	0.414	6.80×10^{-1}	9.78×10^{-1}	0.474
Random70	−0.389	6.98×10^{-1}	9.88×10^{-1}	0.475
Homogeneity	−0.376	7.08×10^{-1}	9.88×10^{-1}	0.471
Random76	0.357	7.22×10^{-1}	9.88×10^{-1}	0.475
Uniformity	0.355	7.24×10^{-1}	9.88×10^{-1}	0.475
Skewness	0.335	7.39×10^{-1}	9.88×10^{-1}	0.474
Random3	−0.329	7.43×10^{-1}	9.88×10^{-1}	0.473
Random28	0.324	7.47×10^{-1}	9.88×10^{-1}	0.473
Random29	0.321	7.49×10^{-1}	9.88×10^{-1}	0.474
Random42	−0.291	7.72×10^{-1}	9.88×10^{-1}	0.474
Random6	0.276	7.83×10^{-1}	9.88×10^{-1}	0.473
Random31	0.269	7.89×10^{-1}	9.88×10^{-1}	0.473
Random23	−0.259	7.97×10^{-1}	9.88×10^{-1}	0.474
Random75	−0.232	8.18×10^{-1}	9.88×10^{-1}	0.474
Random94	0.23	8.19×10^{-1}	9.88×10^{-1}	0.473
Random54	0.226	8.22×10^{-1}	9.88×10^{-1}	0.473

Random98	−0.206	8.37×10^{-1}	9.88×10^{-1}	0.473
Random97	−0.202	8.40×10^{-1}	9.88×10^{-1}	0.473
Random71	0.189	8.50×10^{-1}	9.88×10^{-1}	0.473
Random11	−0.186	8.53×10^{-1}	9.88×10^{-1}	0.473
Random52	0.182	8.56×10^{-1}	9.88×10^{-1}	0.472
Random41	−0.177	8.60×10^{-1}	9.88×10^{-1}	0.473
SZHGE	0.173	8.63×10^{-1}	9.88×10^{-1}	0.473
Random16	−0.165	8.69×10^{-1}	9.88×10^{-1}	0.474
Random67	−0.142	8.88×10^{-1}	9.88×10^{-1}	0.472
Random65	0.138	8.90×10^{-1}	9.88×10^{-1}	0.473
Random39	−0.136	8.92×10^{-1}	9.88×10^{-1}	0.473
Random86	−0.132	8.96×10^{-1}	9.88×10^{-1}	0.472
SZE	−0.125	9.01×10^{-1}	9.88×10^{-1}	0.472
LRHGE	0.121	9.04×10^{-1}	9.88×10^{-1}	0.473
Random82	−0.116	9.08×10^{-1}	9.88×10^{-1}	0.473
Random60	0.114	9.09×10^{-1}	9.88×10^{-1}	0.472
Random4	0.1	9.21×10^{-1}	9.88×10^{-1}	0.473
Random56	−0.096	9.24×10^{-1}	9.88×10^{-1}	0.473
Random34	0.079	9.37×10^{-1}	9.95×10^{-1}	0.473
Random18	0.056	9.56×10^{-1}	9.97×10^{-1}	0.472
Random53	0.048	9.62×10^{-1}	9.97×10^{-1}	0.473
Random21	0.042	9.67×10^{-1}	9.97×10^{-1}	0.473
Dissimilarity	−0.035	9.72×10^{-1}	9.97×10^{-1}	0.472
ZP	0.012	9.90×10^{-1}	9.97×10^{-1}	0.472
Random59	0.011	9.91×10^{-1}	9.97×10^{-1}	0.473
Contrast	0.009	9.93×10^{-1}	9.97×10^{-1}	0.472
Random1	0.004	9.97×10^{-1}	9.97×10^{-1}	0.473

Table S5. Key to radiomics feature nomenclature.

Type of Feature	Type	Feature	Further Description	[Unabbreviated Name: Used in Supp. Material Figures and often in LIFEx v 3.40]
Shape	Shape	Sphericity		SHAPE_Sphericity_LIFEx_5_87
	Shape	Volume		SHAPE_Volume_mL_
Histogram	Histogram	Energy		EnergyH
	Histogram	Entropy_h	Histogram entropy	EntropyH
	Histogram	Kurtosis		KurtosisH
	Histogram	Mean		meanValue
	Histogram	Skewness		SkewnessH
	Histogram	StdDev	Histogram standard deviation	stdValue
Gray-level co-occurrence matrix	GLCM	Contrast		GLCM_Contrast
	GLCM	Correlation		GLCM_Correlation
	GLCM	Dissimilarity		GLCM_Dissimilarity
	GLCM	Uniformity		GLCM_Energy
	GLCM	Entropy		GLCM_Entropy_log10
	GLCM	Homogeneity		GLCM_Homogeneity
Gray-level run-length matrix	GLRLM	GLNUr	Gray-level non-uniformity (runs)	GLRLM_GLNU
	GLRLM	HGRE	High gray-level run emphasis	GLRLM_HGRE
	GLRLM	LGRE	Low gray-level run emphasis	GLRLM_LGRE
	GLRLM	LRE	Long-run emphasis	GLRLM_LRE
	GLRLM	LRHGE	Long-run high gray-level run emphasis	GLRLM_LRHGE
	GLRLM	LRLGE	Long-run low gray-level run emphasis	GLRLM_LRLGE
	GLRLM	RLNU	Run length non-uniformity	GLRLM_RLNU
	GLRLM	RP	Run percentage	GLRLM_RP
	GLRLM	SRE	Short-run emphasis	GLRLM_SRE
	GLRLM	SRHGE	Short-run high gray-level run emphasis	GLRLM_SRHGE
	GLRLM	SRLGE	Short-run low gray-level run emphasis	GLRLM_SRLGE
Gray-level zone-length matrix	GLZLM	GLNUz	Gray-level non-uniformity (zones)	GLZLM_GLNU
	GLZLM	HGZE	High gray-level zone emphasis	GLZLM_HGZE
	GLZLM	LGZE	Low gray-level zone emphasis	GLZLM_LGZE
	GLZLM	LZE	Long zone emphasis	GLZLM_LZE
	GLZLM	LZHGE	Long zone high gray-level run emphasis	GLZLM_LZHGE

	GLZLM	LZLGE	Long zone low gray-level run emphasis	GLZLM_LZLGE
	GLZLM	SZE	Short zone emphasis	GLZLM_SZE
	GLZLM	SZHGE	Short zone high gray-level run emphasis	GLZLM_SZHGE
	GLZLM	SZLGE	Short zone low gray-level run emphasis	GLZLM_SZLGE
	GLZLM	ZLNU	Zone-length non-uniformity	GLZLM_ZLNU
	GLZLM	ZP	Zone percentage	GLZLM_ZP
Neighborhood gray-level difference matrix	NGLDM	Busyness		NGLDM_Busyness
	NGLDM	Coarseness		NGLDM_Coarseness
	NGLDM	Contrast		NGLDM_Contrast
